# Supplementary material for: Role of distinct fibroblast lineages and immune cells in dermal repair following UV radiation-induced tissue damage
Source: eLife. 2021 Dec 23;10:e71052. doi: 10.7554/eLife.71052 (PMC8747514; doi:10.7554/eLife.71052)
Supplement: Figure 1—figure supplement 2—source data 1. — Files include the analysis protocol files of P2 and P21 mouse skin single-cell RNA-seq datasets and GO term enrichment analysis of indicated datasets and figure panel. [file elife-71052-fig1-figsupp2-data1.zip › Figure 1- supplementary Souce file 2/Figure S2A - P2 UV analysis.pdf]

P2 UV analysis

November 19, 2021

[1]:
```
import os
import numpy as np
import pandas as pd
os.environ['PATH'] += os.pathsep + os.path.
 ↪expanduser('~\\AppData\\Roaming\\Python\\Lib\\site-packages\\tables')
import scanpy as sc
import loompy as lp
#from MulticoreTSNE import MulticoreTSNE as TSNE
import json
import base64
import zlib
from pyscenic.plotting import plot_binarization
#from pyscenic.export import add_scenic_metadata
#from pyscenic.cli.utils import load_signatures
import matplotlib as mpl
import matplotlib.pyplot as plt
#from scanpy.plotting._tools.scatterplots import plot_scatter
#import scanpy.pl.scatter as plot_scatter
import seaborn as sns
```

[2]:
```
adata = sc.read( 'all_cells.loom', validate=False)
```

```
Variable names are not unique. To make them unique, call
`.var_names_make_unique`.
```

[3]:
```
adata.var_names_make_unique()
```

[6]:
```
adata = adata[adata.obs.age==2]
```

[8]:
```
# Set up some scanpy preferences
sc.settings.verbosity = 3 # verbosity: errors (0), warnings (1), info (2),␣
 ↪hints (3)
#sc.logging.print_versions()
sc.set_figure_params(dpi=120, fontsize=10, dpi_save=600)

# Set maximum number of jobs for Scanpy. Stops the computer freezing up using␣
 ↪all the cores
sc.settings.njobs = 4
```

[9]:
```
sc.pl.highest_expr_genes(adata, n_top=20, )
```

```
C:\Users\hhy696\OneDrive - Queen Mary, University of
London\Bioinformatics\bioinf\lib\site-
packages\scanpy\preprocessing\_normalization.py:155: UserWarning: Revieved a
view of an AnnData. Making a copy.
  view_to_actual(adata)
```

```
normalizing counts per cell
    finished (0:00:00)
```

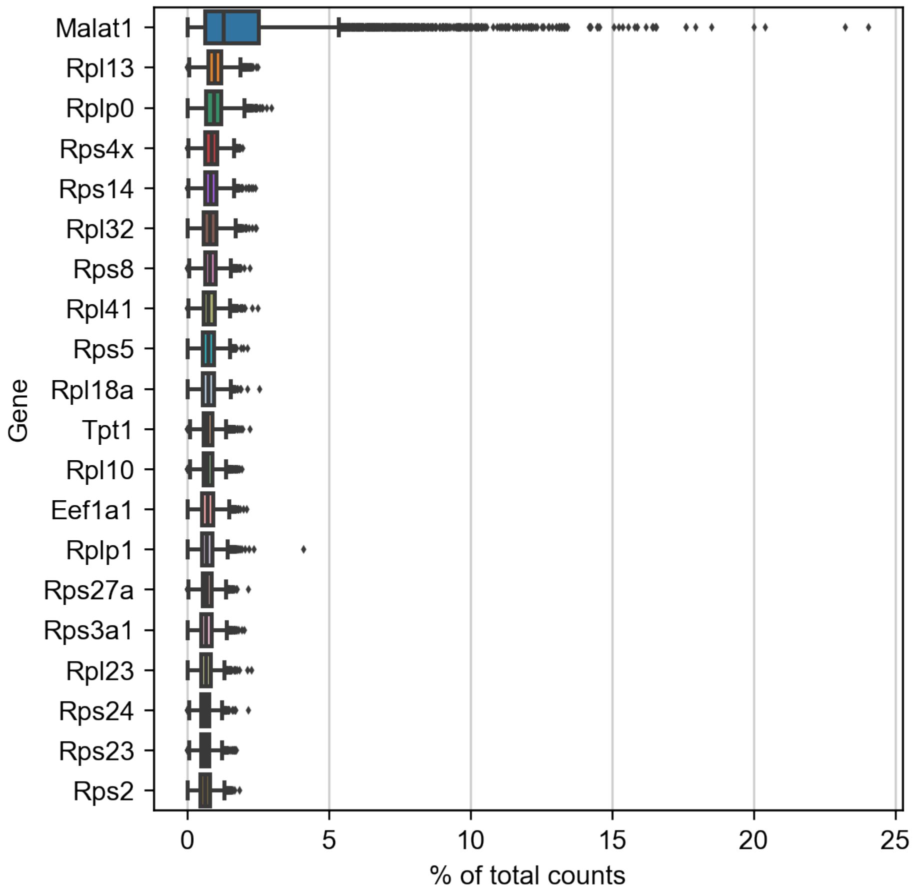

[10]:
```
# filter cells with less than 200 genes expressed or genes expressed in less␣
↪than 3 cells
sc.pp.filter_cells(adata, min_genes=200)
sc.pp.filter_genes(adata, min_cells=3)
```

```
filtered out 43 cells that have less than 200 genes expressed
filtered out 13773 genes that are detected in less than 3 cells
```

[11]:
```
sc.pp.calculate_qc_metrics(adata, percent_top=None, log1p=False, inplace=True)
```

[12]:
```
sc.pl.violin(adata, ['n_genes_by_counts', 'total_counts'],
             jitter=0.4, multi_panel=True)
```

```
C:\Users\hhy696\OneDrive - Queen Mary, University of
London\Bioinformatics\bioinf\lib\site-packages\anndata\_core\anndata.py:1220:
FutureWarning: The `inplace` parameter in pandas.Categorical.reorder_categories
is deprecated and will be removed in a future version. Removing unused
categories will always return a new Categorical object.
  c.reorder_categories(natsorted(c.categories), inplace=True)
… storing 'CreationDate' as categorical
C:\Users\hhy696\OneDrive - Queen Mary, University of
London\Bioinformatics\bioinf\lib\site-packages\anndata\_core\anndata.py:1220:
FutureWarning: The `inplace` parameter in pandas.Categorical.reorder_categories
is deprecated and will be removed in a future version. Removing unused
categories will always return a new Categorical object.
  c.reorder_categories(natsorted(c.categories), inplace=True)
… storing 'last_modified' as categorical
C:\Users\hhy696\OneDrive - Queen Mary, University of
London\Bioinformatics\bioinf\lib\site-packages\anndata\_core\anndata.py:1220:
FutureWarning: The `inplace` parameter in pandas.Categorical.reorder_categories
is deprecated and will be removed in a future version. Removing unused
categories will always return a new Categorical object.
  c.reorder_categories(natsorted(c.categories), inplace=True)
… storing 'Chromosome' as categorical
C:\Users\hhy696\OneDrive - Queen Mary, University of
London\Bioinformatics\bioinf\lib\site-packages\anndata\_core\anndata.py:1220:
FutureWarning: The `inplace` parameter in pandas.Categorical.reorder_categories
is deprecated and will be removed in a future version. Removing unused
categories will always return a new Categorical object.
  c.reorder_categories(natsorted(c.categories), inplace=True)
… storing 'Strand' as categorical
```

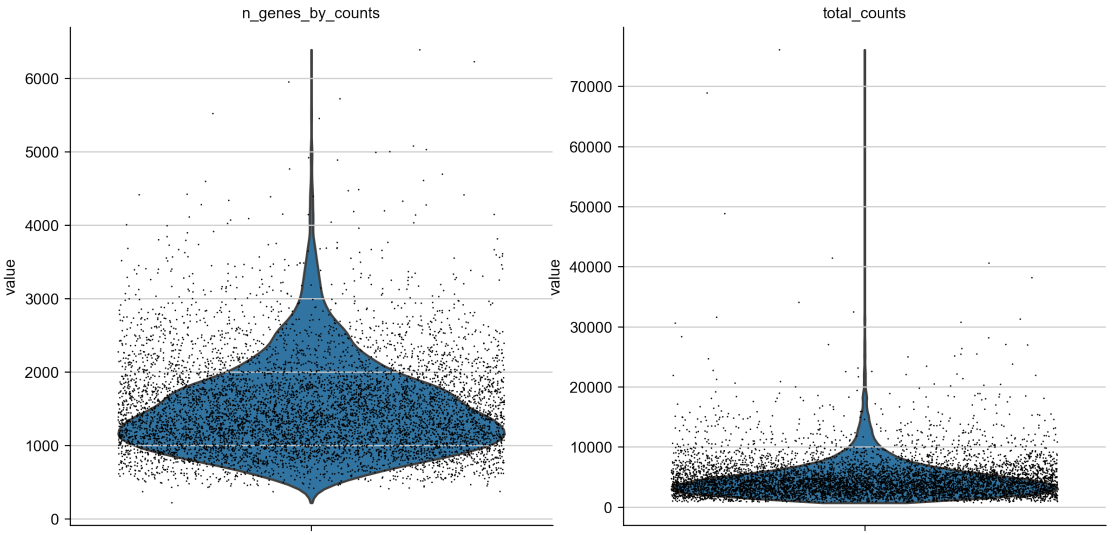

[13]:
```
sc.pl.scatter(adata, x='total_counts', y='n_genes_by_counts')
```

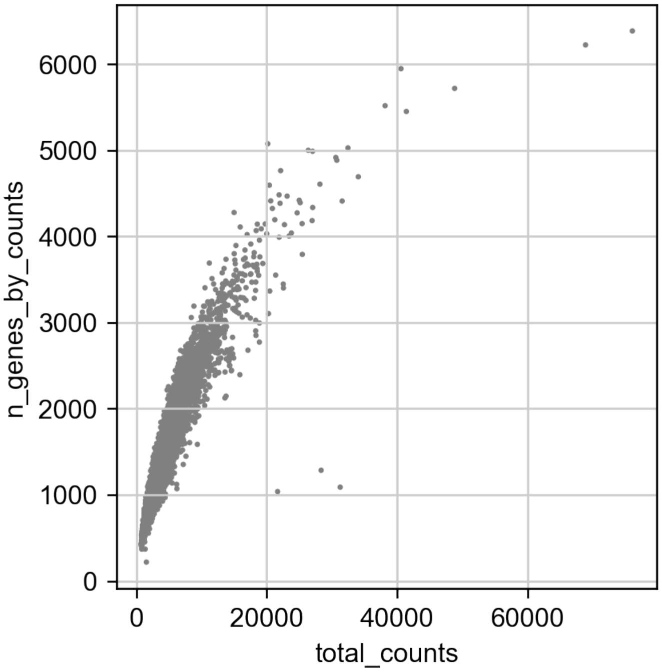

[14]:
```
# Do some more preprocessing https://scanpy-tutorials.readthedocs.io/en/latest/
↪pbmc3k.html
sc.pp.normalize_total(adata, target_sum=1e4)
sc.pp.log1p(adata)
sc.pp.highly_variable_genes(adata, min_mean=0.0125, max_mean=3, min_disp=0.5)
sc.pl.highly_variable_genes(adata)
```

```
normalizing counts per cell
    finished (0:00:00)
extracting highly variable genes
    finished (0:00:00)
--> added
    'highly_variable', boolean vector (adata.var)
    'means', float vector (adata.var)
    'dispersions', float vector (adata.var)
    'dispersions_norm', float vector (adata.var)
```

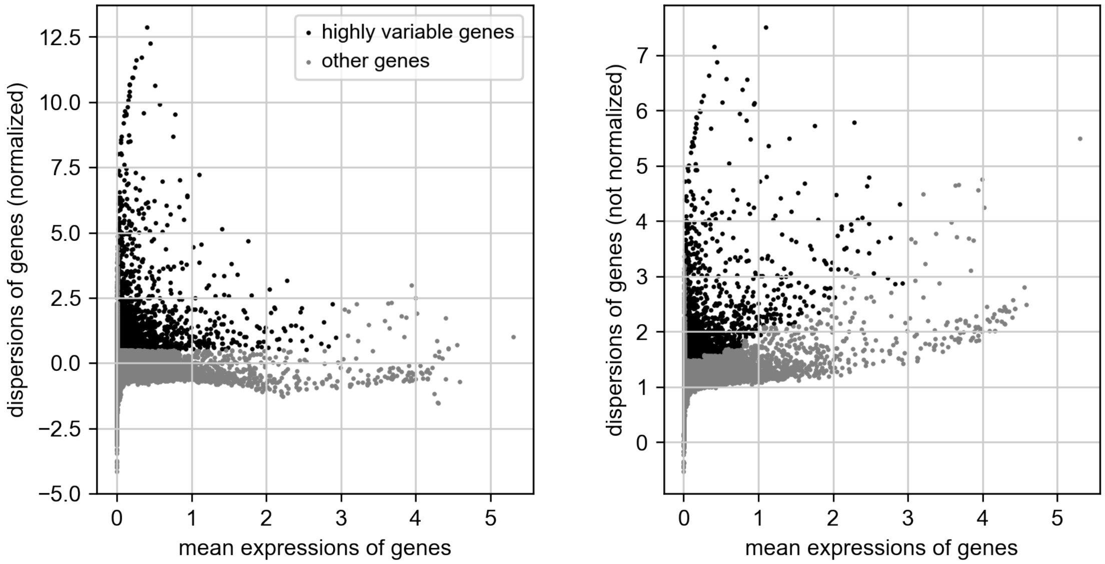

[15]:
```
# save a copy of the raw data. This is sometimes used by other functions later
adata.raw = adata
```

[16]:
```
# more preprocessing
adata = adata[:, adata.var.highly_variable]
sc.pp.regress_out(adata, ['total_counts'])
sc.pp.scale(adata, max_value=10)
```

```
regressing out ['total_counts']
    sparse input is densified and may lead to high memory use
    finished (0:00:15)
```

[17]:
```
# run principal component analysis
sc.tl.pca(adata, n_comps=30)
```

```
computing PCA
    on highly variable genes
    with n_comps=30
    finished (0:00:01)
```

[19]:
```
# plot the PCA, colouring by wounded and some fibroblast genes
sc.pl.pca(adata, color=['age', 'sample', 'Pdgfra', 'Twist2', 'Col1a2'], alpha=0.
↪5)
```

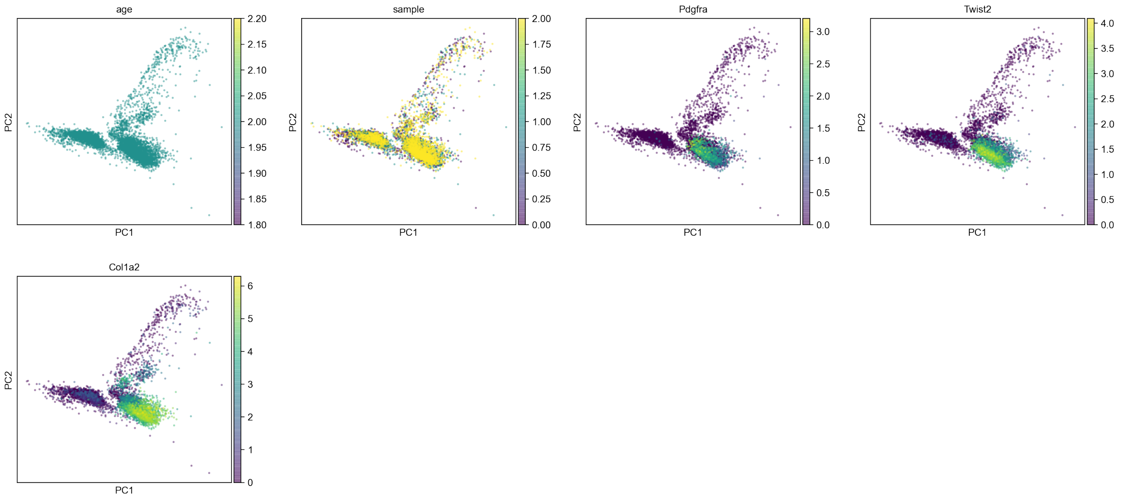


```
[20]: adata.obs['sample'] = adata.obs['sample'].astype('category')
```

```
[21]: # plot the contribution of each PC to the variance
      sc.pl.pca_variance_ratio(adata, log=True)
```

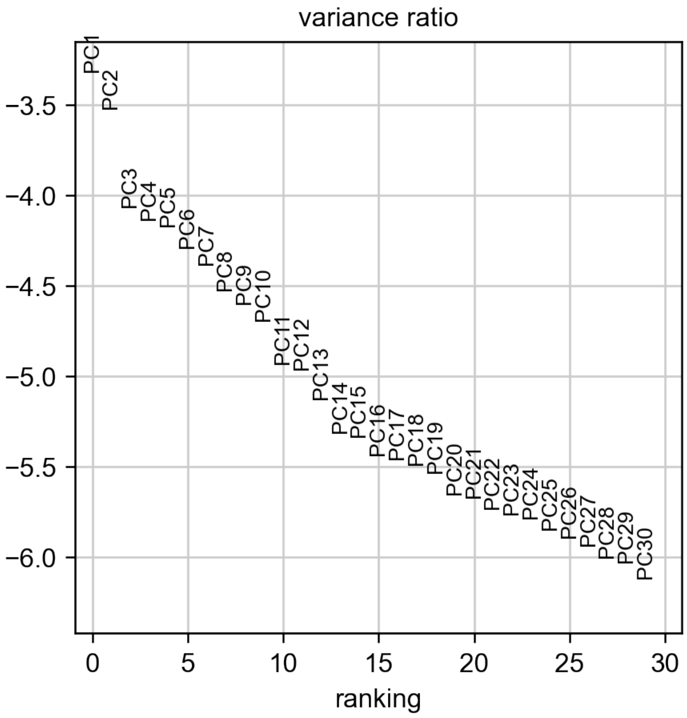

[22]:
```
# write your filtered dataset
adata.write('P2 cells filtered.h5ad', compression='lzf')
```

[23]:
```
# compute umap embedding
sc.pp.neighbors(adata, n_neighbors=10)
sc.tl.umap(adata)
sc.pl.umap(adata, color=['sample', 'age', 'Pdgfra', 'Twist2', 'Col1a2'])
```

```
computing neighbors
    using 'X_pca' with n_pcs = 30
    finished: added to `.uns['neighbors']`
    `.obsp['distances']`, distances for each pair of neighbors
    `.obsp['connectivities']`, weighted adjacency matrix (0:00:05)
computing UMAP
    finished: added
    'X_umap', UMAP coordinates (adata.obsm) (0:00:11)
```

```
---------------------------------------------------------------------------
KeyError                                  Traceback (most recent call last)
~\OneDrive - Queen Mary, University of␣
 ↪London\Bioinformatics\bioinf\lib\site-packages\pandas\core\indexes\base.py in␣
 ↪get_loc(self, key, method, tolerance)
   3360             try:
-> 3361                 return self._engine.get_loc(casted_key)
   3362             except KeyError as err:

~\OneDrive - Queen Mary, University of␣
 ↪London\Bioinformatics\bioinf\lib\site-packages\pandas\_libs\index.pyx in␣
 ↪pandas._libs.index.IndexEngine.get_loc()

~\OneDrive - Queen Mary, University of␣
 ↪London\Bioinformatics\bioinf\lib\site-packages\pandas\_libs\index.pyx in␣
 ↪pandas._libs.index.IndexEngine.get_loc()

pandas\_libs\hashtable_class_helper.pxi in pandas._libs.hashtable.
 ↪PyObjectHashTable.get_item()

pandas\_libs\hashtable_class_helper.pxi in pandas._libs.hashtable.
 ↪PyObjectHashTable.get_item()

KeyError: 'wounded'

The above exception was the direct cause of the following exception:

KeyError                                  Traceback (most recent call last)
~\AppData\Local\Temp/ipykernel_9132/1179834150.py in <module>
      2 sc.pp.neighbors(adata, n_neighbors=10)
      3 sc.tl.umap(adata)
```

```
----> 4 sc.pl.umap(adata, color=['sample', 'wounded', 'Pdgfra', 'Twist2',␣
 ↪'Col1a2'])

~\OneDrive - Queen Mary, University of␣
 ↪London\Bioinformatics\bioinf\lib\site-packages\scanpy\plotting\_tools\scatterplots.
 ↪py in umap(adata, **kwargs)
    657     tl.umap
    658     """
--> 659     return embedding(adata, 'umap', **kwargs)
    660
    661

~\OneDrive - Queen Mary, University of␣
 ↪London\Bioinformatics\bioinf\lib\site-packages\scanpy\plotting\_tools\scatterplots.
 ↪py in embedding(adata, basis, color, gene_symbols, use_raw, sort_order, edges, ␣
 ↪edges_width, edges_color, neighbors_key, arrows, arrows_kwds, groups,␣
 ↪components, layer, projection, scale_factor, color_map, cmap, palette,␣
 ↪na_color, na_in_legend, size, frameon, legend_fontsize, legend_fontweight,␣
 ↪legend_loc, legend_fontoutline, vmax, vmin, vcenter, norm, add_outline,␣
 ↪outline_width, outline_color, ncols, hspace, wspace, title, show, save, ax,␣
 ↪return_fig, **kwargs)
    242         itertools.product(color, idx_components)
    243     ):
--> 244         color_source_vector = _get_color_source_vector(

    245             adata,
    246             value_to_plot,

~\OneDrive - Queen Mary, University of␣
 ↪London\Bioinformatics\bioinf\lib\site-packages\scanpy\plotting\_tools\scatterplots.
 ↪py in _get_color_source_vector(adata, value_to_plot, use_raw, gene_symbols,␣
 ↪layer, groups)
   1221         ]  # TODO: Throw helpful error if this doesn't work
   1222     if use_raw and value_to_plot not in adata.obs.columns:
-> 1223         values = adata.raw.obs_vector(value_to_plot)
   1224     else:
   1225         values = adata.obs_vector(value_to_plot, layer=layer)

~\OneDrive - Queen Mary, University of␣
 ↪London\Bioinformatics\bioinf\lib\site-packages\anndata\_core\raw.py in␣
 ↪obs_vector(self, k)
    168     def obs_vector(self, k: str) -> np.ndarray:
    169         # TODO decorator to copy AnnData.obs_vector docstring
--> 170         idx = self._normalize_indices((slice(None), k))
    171         a = self.X[idx]
    172         if issparse(a):

~\OneDrive - Queen Mary, University of␣
 ↪London\Bioinformatics\bioinf\lib\site-packages\anndata\_core\raw.py in␣
 ↪_normalize_indices(self, packed_index)
    159         obs, var = unpack_index(packed_index)
```

```
    160         obs = _normalize_index(obs, self._adata.obs_names)
--> 161         var = _normalize_index(var, self.var_names)
    162         return obs, var
    163

~\OneDrive - Queen Mary, University of␣
 ↪London\Bioinformatics\bioinf\lib\site-packages\anndata\_core\index.py in␣
 ↪_normalize_index(indexer, index)
     73         return indexer
     74     elif isinstance(indexer, str):
---> 75         return index.get_loc(indexer)  # int
     76     elif isinstance(indexer, (Sequence, np.ndarray, pd.Index, spmatrix,␣
 ↪np.matrix)):
     77         if hasattr(indexer, "shape") and (

~\OneDrive - Queen Mary, University of␣
 ↪London\Bioinformatics\bioinf\lib\site-packages\pandas\core\indexes\base.py in␣
 ↪get_loc(self, key, method, tolerance)
   3361                 return self._engine.get_loc(casted_key)
   3362             except KeyError as err:
-> 3363                 raise KeyError(key) from err
   3364
   3365         if is_scalar(key) and isna(key) and not self.hasnans:

KeyError: 'wounded'
```

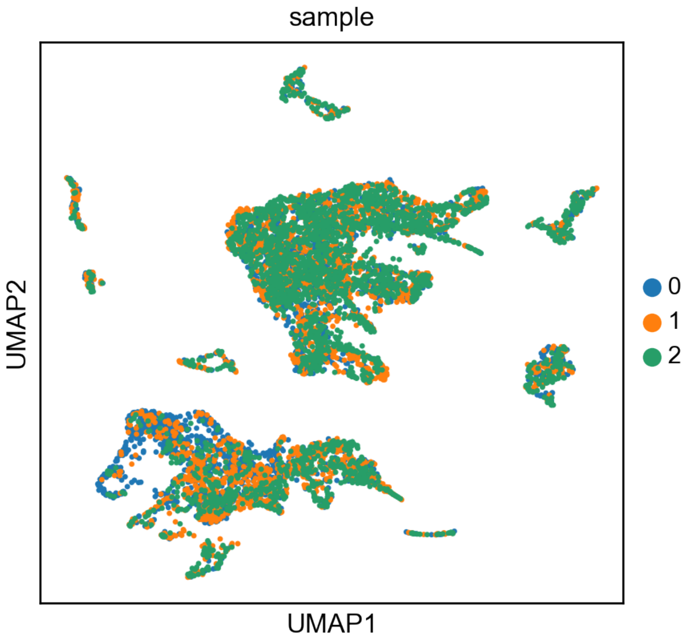

[24]:
```
sc.pl.umap(adata, color=['sample', 'age', 'Pdgfra', 'Twist2', 'Col1a2'])
```

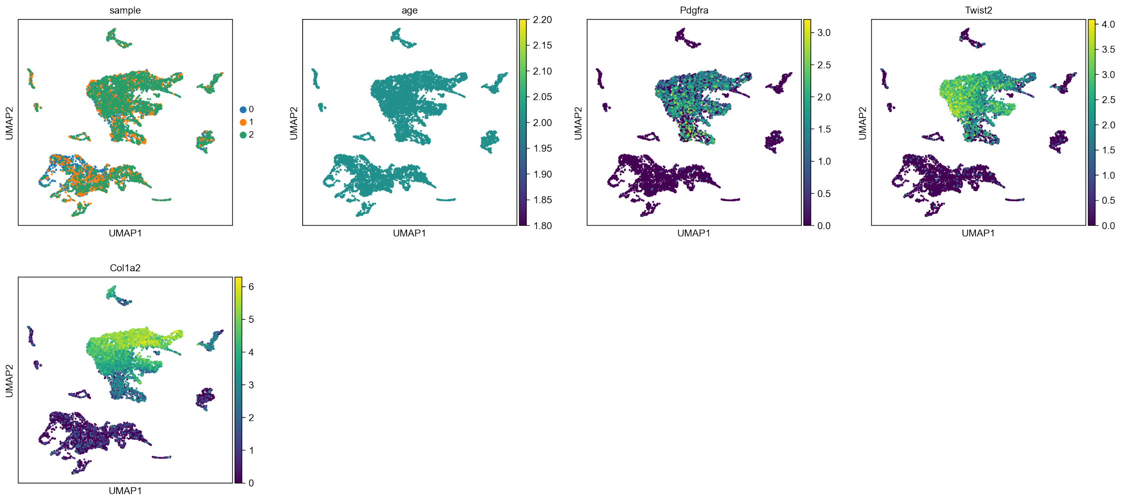

[27]:
```
# cluster the data
sc.tl.leiden(adata, resolution=0.2)
sc.pl.umap(adata, color=['leiden'])
```

```
running Leiden clustering
    finished: found 15 clusters and added
    'leiden', the cluster labels (adata.obs, categorical) (0:00:00)
```

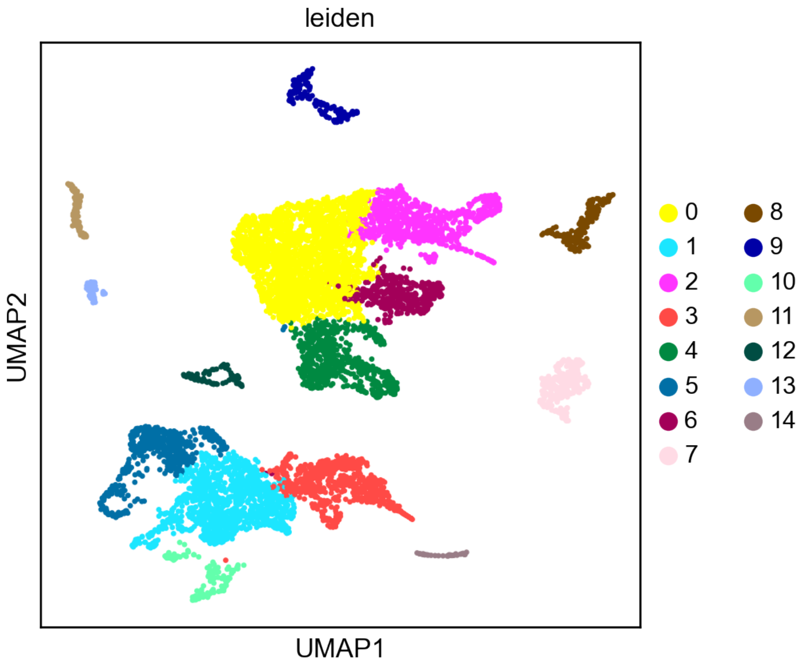

[29]:
```
sc.pl.umap(adata, color=['En1', 'Pdgfra', 'Twist2'] ) # fibroblasts
sc.pl.umap(adata, color=['Col1a1', 'Vim', 'Thy1'] )
sc.pl.umap(adata, color=['Krt10', 'Krt14'] ) # keratinocytes
sc.pl.umap(adata, color=['Pecam1', 'Ly6e', 'Ecscr'] ) # endothelial (Ly6e+) and lymphatic (Ly6e-)
sc.pl.umap(adata, color=['Laptm5', 'Cd53', 'Coro1a'] )# immune cells
sc.pl.umap(adata, color=['Rgs5', 'Mylk'] )              # pericytes
sc.pl.umap(adata, color=['Dct', 'Ptgds'] )              # melanocytes
sc.pl.umap(adata, color=['Plp1', 'Dbi'] )               # Schwann cells (developing)
sc.pl.umap(adata, color=['Etv1', 'Fth1'] )              # Scwann cells (homeostasis)
```

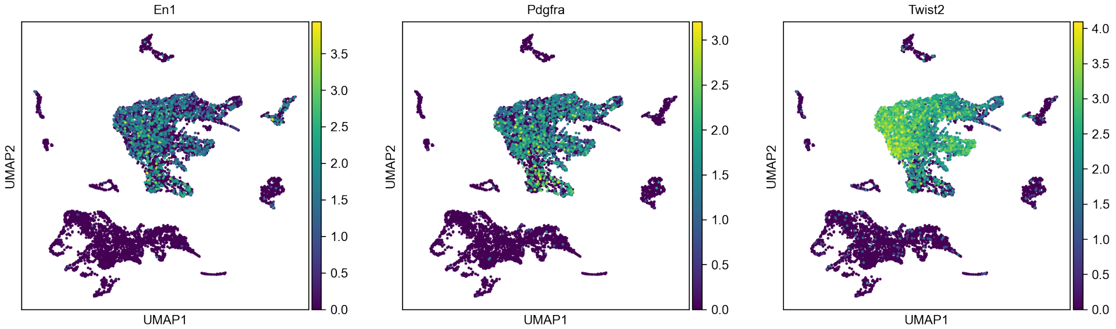

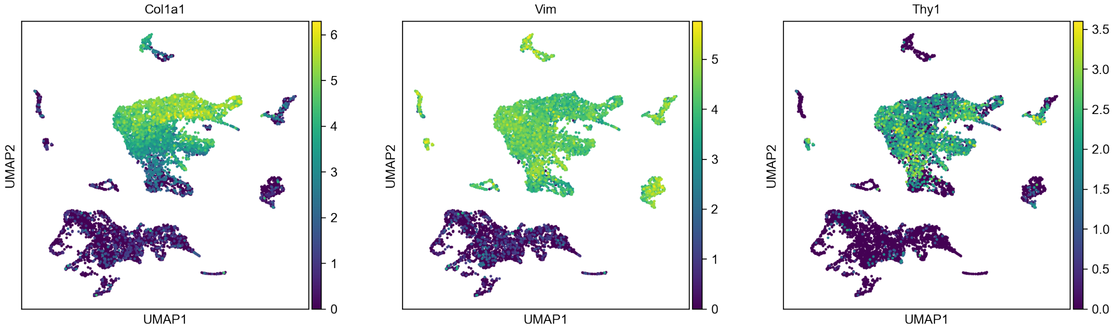

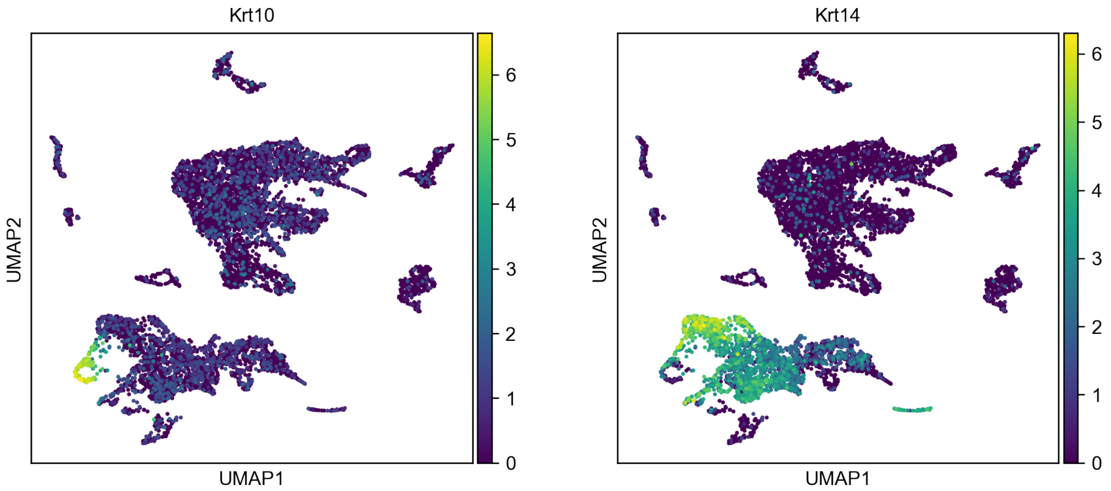
Krt10
Krt14
UMAP2
UMAP1

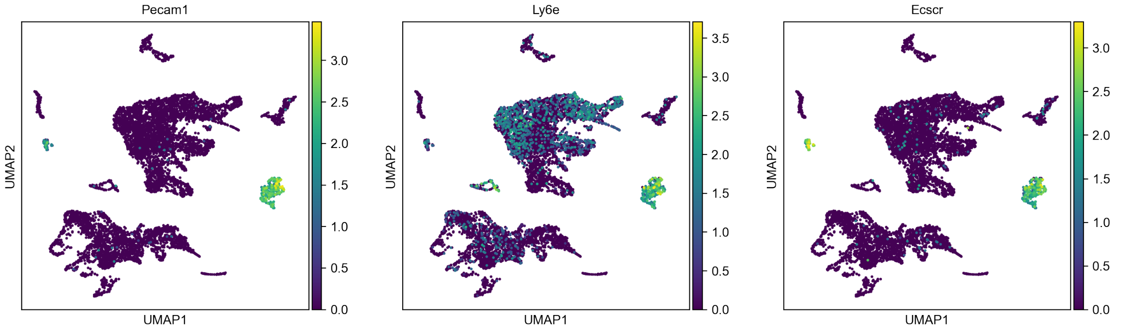
Pecam1
Ly6e
Ecscr
UMAP2
UMAP1

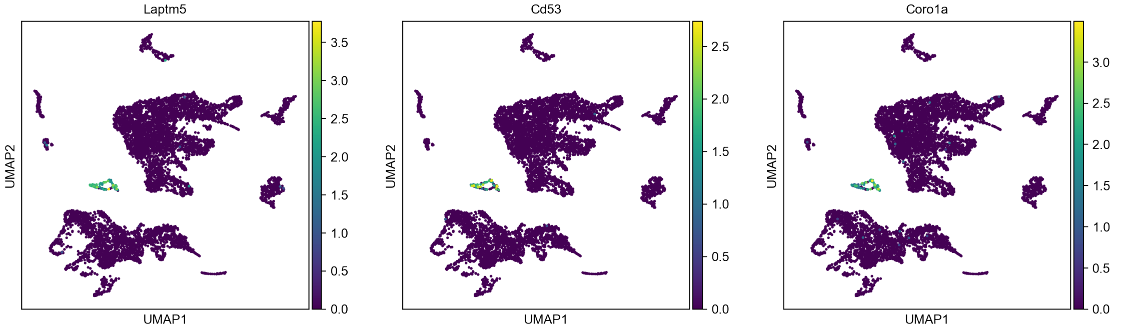
Laptm5
Cd53
Coro1a
UMAP2
UMAP1

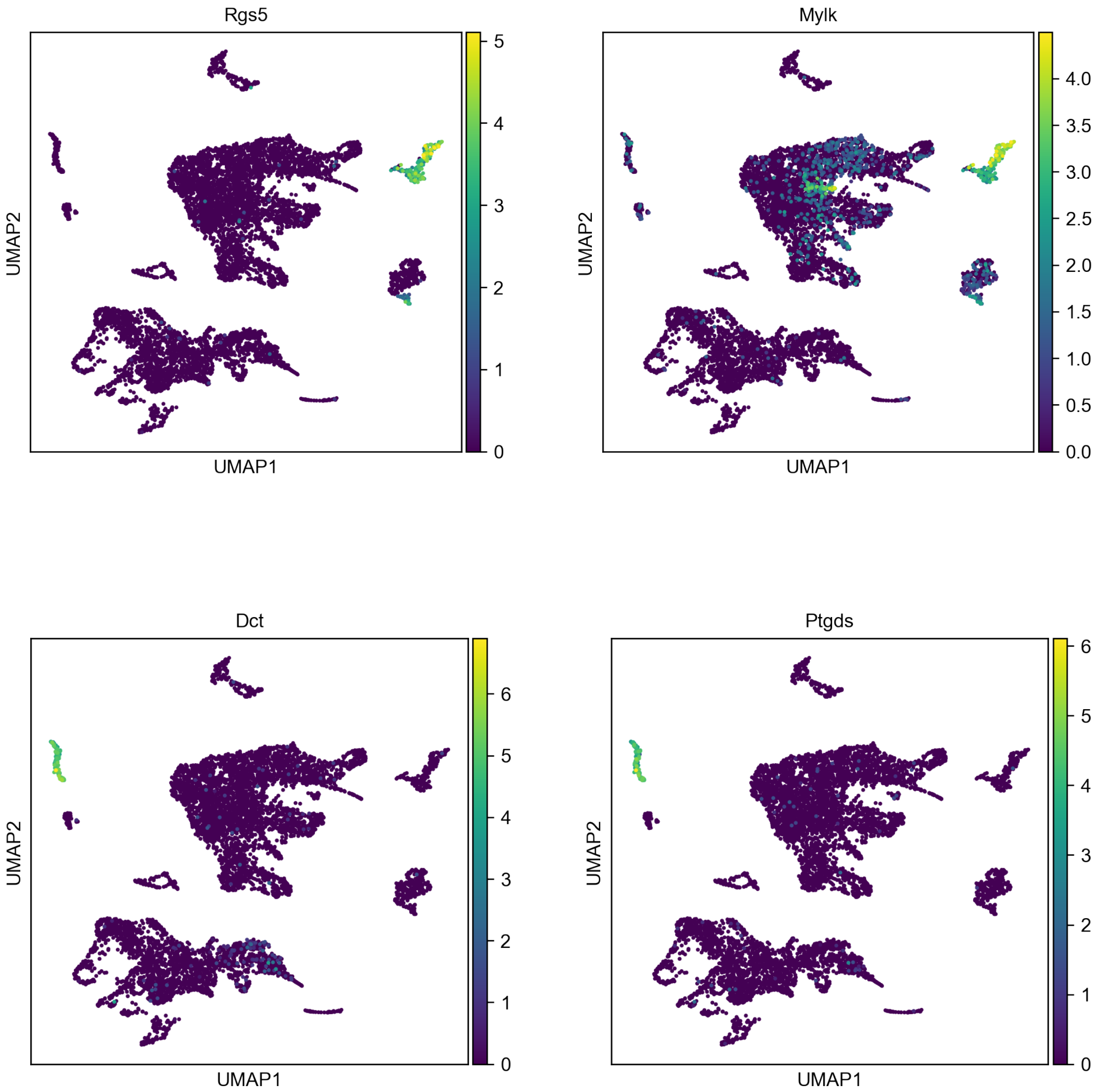
Rgs5
Mylk
Dct
Ptgds
UMAP1
UMAP2
0 1 2 3 4 5
0.0 0.5 1.0 1.5 2.0 2.5 3.0 3.5 4.0
0 1 2 3 4 5 6
0 1 2 3 4 5 6

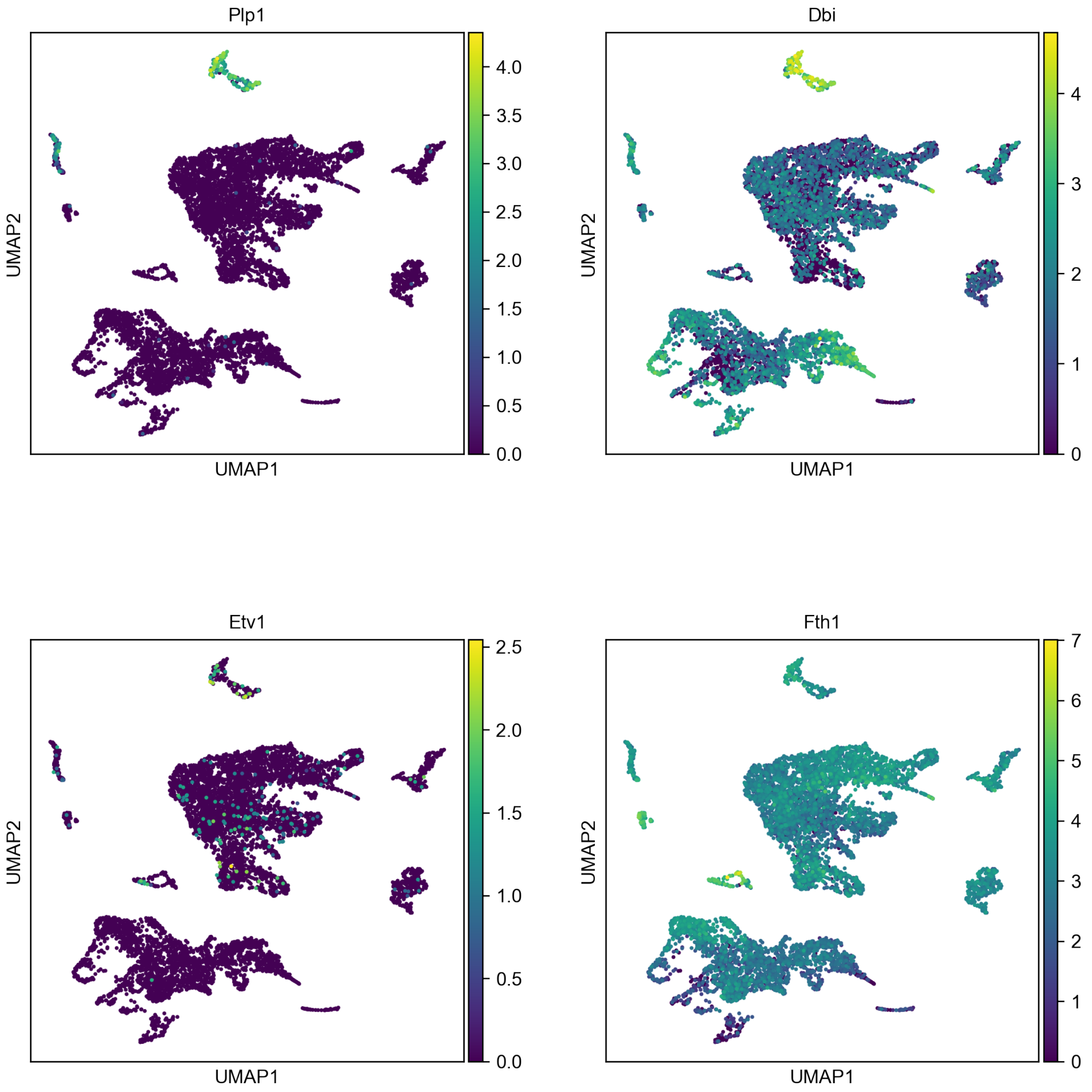


```
[30]: main_clusters =␣
       ↪['Fibroblast','Keratinocyte','Fibroblast','Keratinocyte','Fibroblast','Keratinocyte','Fibro
                        'Endothelial','SMC/
       ↪pericyte','Schwann','','Melanocyte','Immune','Endothelial','']
      fibroblast_clusters = ['0','2','4','6']
      sc.pl.umap(adata[adata.obs.leiden.isin(fibroblast_clusters)], color='leiden')
```

```
Trying to set attribute `.uns` of view, copying.
```

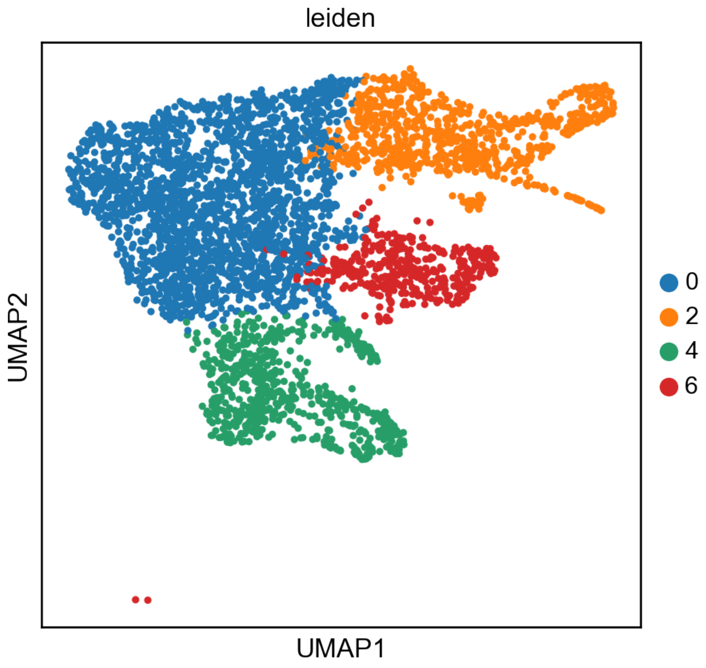

[31]:
```
fbs = adata[adata.obs.leiden.isin(fibroblast_clusters)]
fbs
```

[31]:
```
View of AnnData object with n_obs × n_vars = 3961 × 1874
    obs: 'Clusters', 'CreationDate', '_X', '_Y', 'age', 'last_modified',
'sample', 'n_genes', 'n_genes_by_counts', 'total_counts', 'leiden'
    var: 'Accession', 'Chromosome', 'End', 'Start', 'Strand', 'n_cells',
'n_cells_by_counts', 'mean_counts', 'pct_dropout_by_counts', 'total_counts',
'highly_variable', 'means', 'dispersions', 'dispersions_norm', 'mean', 'std'
    uns: 'log1p', 'hvg', 'pca', 'neighbors', 'umap', 'sample_colors', 'leiden'
    obsm: 'X_pca', 'X_umap'
    varm: 'PCs'
    layers: 'matrix', 'ambiguous', 'spliced', 'unspliced'
    obsp: 'distances', 'connectivities'
```

[32]:
```
fbs.write('P2 fibroblasts.h5ad', compression='lzf')
```

[34]:
```
# delete all old attributes so we can recompute umap
del fbs.uns['neighbors']
del fbs.uns['pca']
del fbs.obsp['distances']
del fbs.obsp['connectivities']
del fbs.obsm['X_pca']
del fbs.obsm['X_umap']
del fbs.varm['PCs']
```

```
# compute umap embedding
sc.pp.neighbors(fbs, n_neighbors=10, n_pcs=40)
sc.tl.umap(fbs)
sc.pl.umap(fbs)
```

```
computing neighbors
WARNING: You’re trying to run this on 1874 dimensions of `.X`, if you really
want this, set `use_rep='X'`.
         Falling back to preprocessing with `sc.pp.pca` and default params.
computing PCA
    with n_comps=50
    finished (0:00:01)
    finished: added to `.uns['neighbors']`
    `.obsp['distances']`, distances for each pair of neighbors
    `.obsp['connectivities']`, weighted adjacency matrix (0:00:01)
computing UMAP
    finished: added
    'X_umap', UMAP coordinates (adata.obsm) (0:00:08)
```

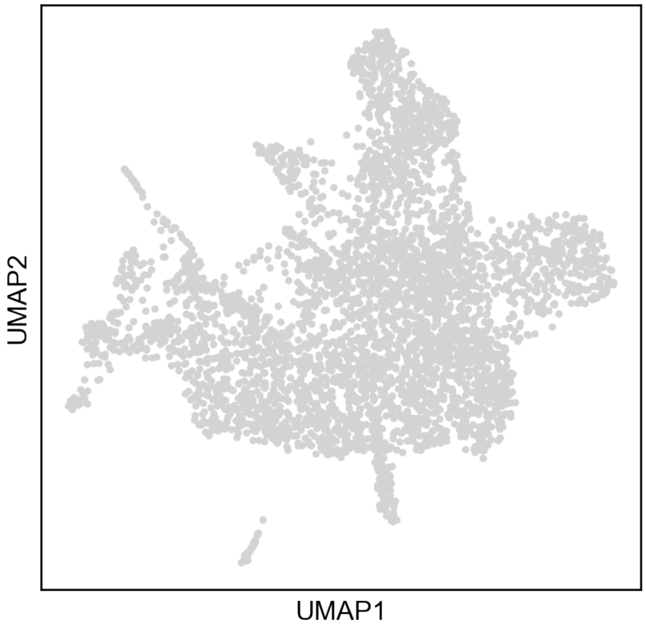

[45]:
```
# cluster the data
sc.tl.leiden(fbs, resolution=0.5)
sc.pl.umap(fbs, color=['leiden'])
```

```
running Leiden clustering
    finished: found 9 clusters and added
```

```
'leiden', the cluster labels (adata.obs, categorical) (0:00:00)
```

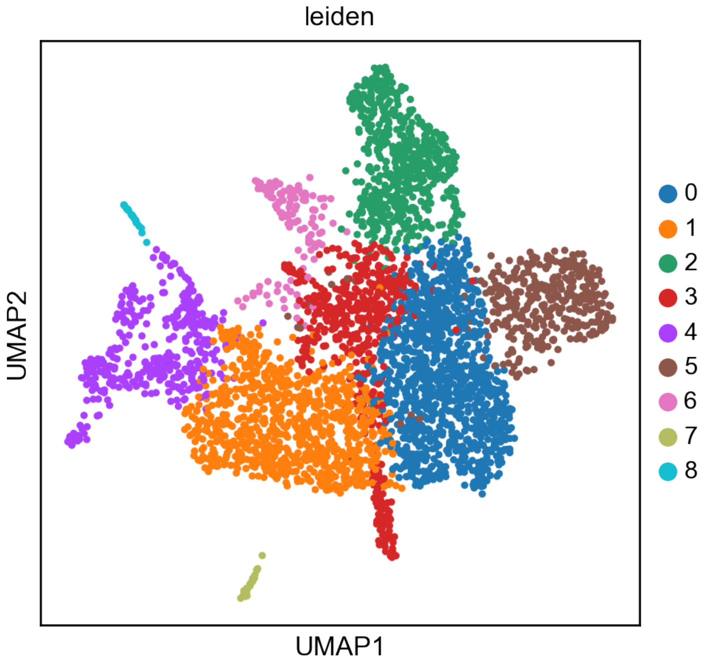

[48]:
```
sc.pl.umap(fbs, color=['leiden','Dpp4','Dlk1', 'Ly6a','Dkk1','Acta2','Corin',␣
↪'Acan','Gpx3'])
```

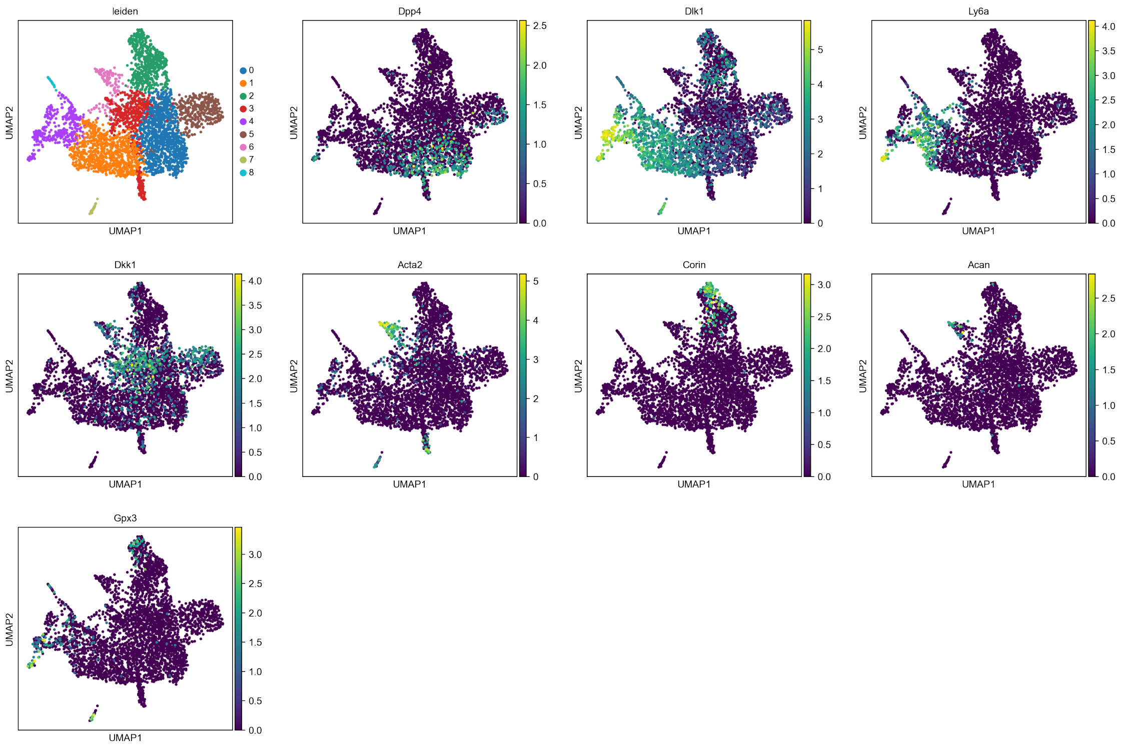

[51]:
```
cluster_names = ['Dpp4 papillary','Reticular','DP','Dkk1 papillary','Preadipocyte', 'Dividin papillary',
                 'APM','Pericyte','Adipocyte']
```

[125]:
```
sc.tl.rank_genes_groups(fbs, 'leiden', method='t-test')
sc.pl.rank_genes_groups(fbs, n_genes=25, sharey=False)
```

```
ranking genes
    finished: added to `.uns['rank_genes_groups']`
    'names', sorted np.recarray to be indexed by group ids
    'scores', sorted np.recarray to be indexed by group ids
    'logfoldchanges', sorted np.recarray to be indexed by group ids
    'pvals', sorted np.recarray to be indexed by group ids
    'pvals_adj', sorted np.recarray to be indexed by group ids (0:00:00)
```

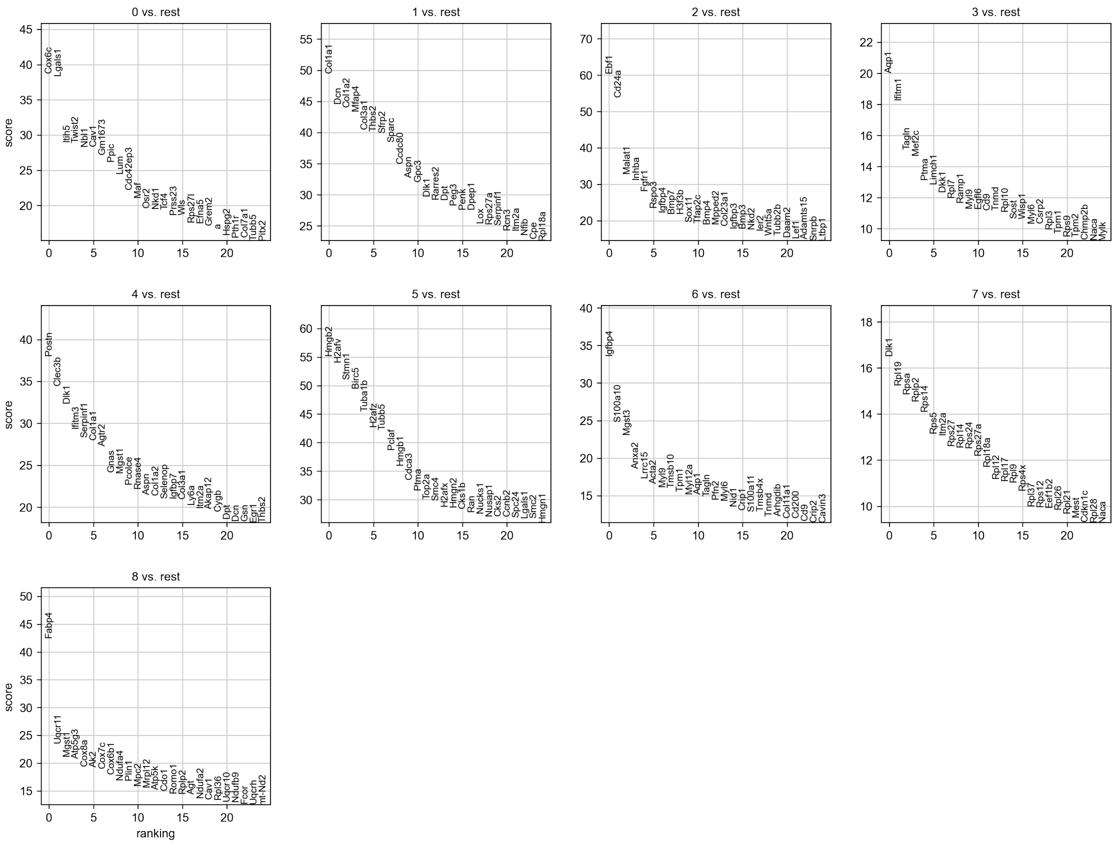

[56]:
```
fbs.obs['cell_type'] = [cluster_names[int(x)] for x in fbs.obs.leiden]
```

[58]:
```
sc.pl.umap(fbs, color=['cell_type'])
sc.pl.umap(fbs, color=['Dpp4','Dlk1', 'Ly6a','Dkk1','Acta2','Corin',␣
↪'Acan','Gpx3'])
```

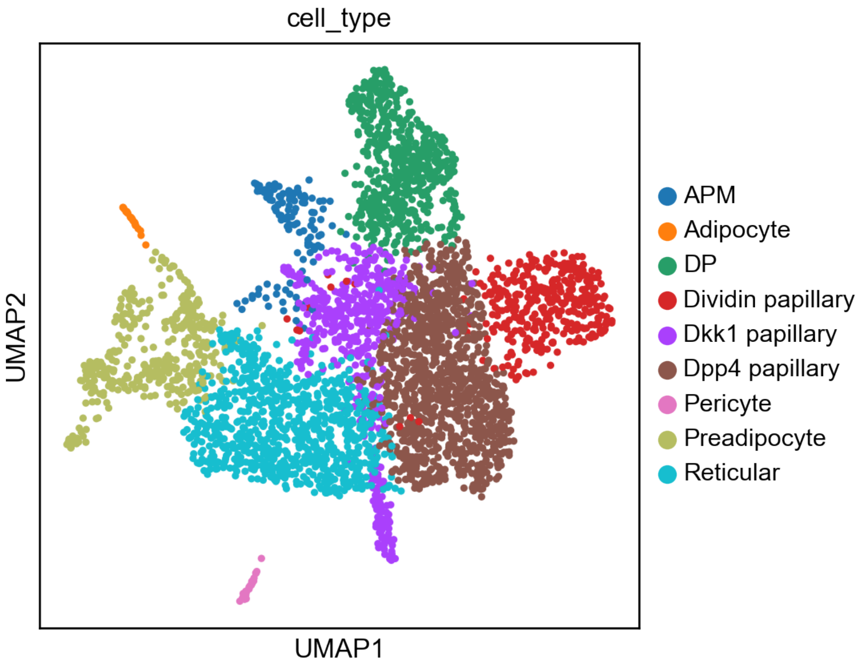

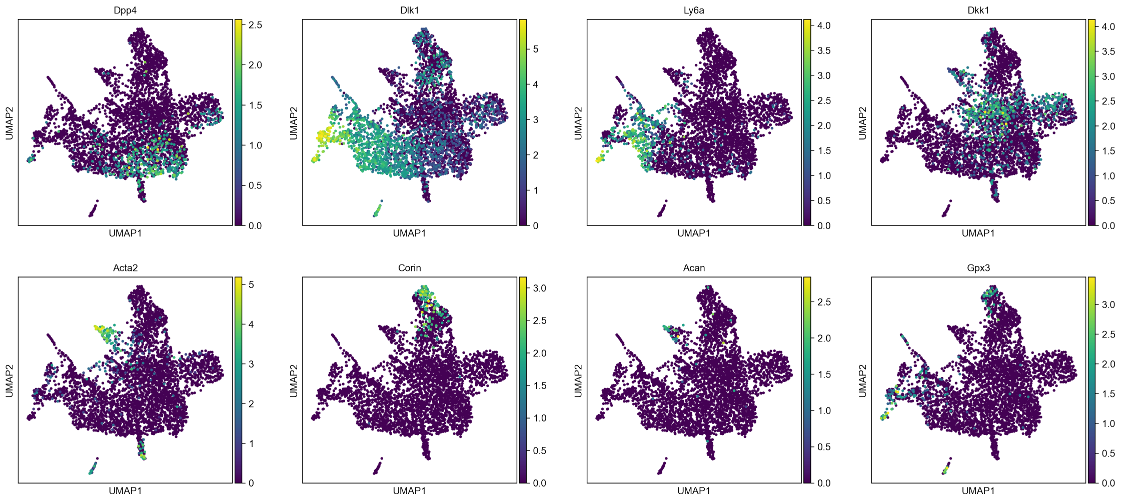

[60]:
```
sc.pl.umap(fbs, color=['Dpp4','Dlk1', 'Ly6a','Dkk1','Stmn2','Corin',
 ↪'Acan','Gpx3'])
```

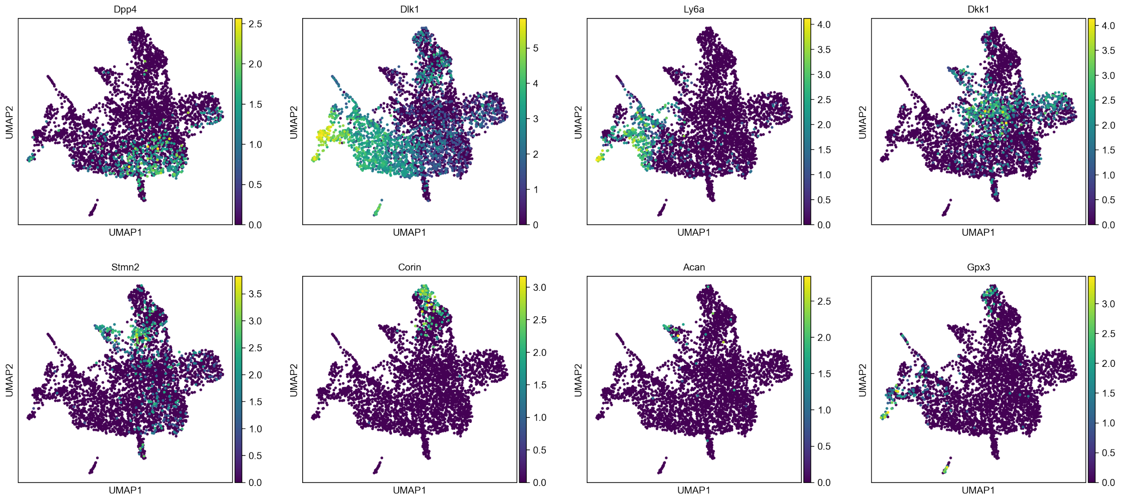

[71]:
```
dpp4_pap = sc.queries.enrich(fbs, '0', gprofiler_kwargs={'all_results':True,
 ↪'sources':['GO:BP']})
```

[63]:
```
fbs.write('P2 fibroblasts.h5ad', compression='lzf')
```

[73]:
```
cluster_names
```

[73]:
```
['Dpp4 papillary',
 'Reticular',
 'DP',
 'Dkk1 papillary',
 'Preadipocyte',
 'Dividin papillary',
 'APM',
 'Pericyte',
 'Adipocyte']
```

[74]:
```
reticular = sc.queries.enrich(fbs, '1', gprofiler_kwargs={'all_results':True,
 ↪'sources':['GO:BP']})
```

[75]:
```
dkk1_pap = sc.queries.enrich(fbs, '3', gprofiler_kwargs={'all_results':True,
 ↪'sources':['GO:BP']})
```

[76]:
```
preadipocyte = sc.queries.enrich(fbs, '4', gprofiler_kwargs={'all_results':
 ↪True, 'sources':['GO:BP']})
```

[77]:
```
div_pap = sc.queries.enrich(fbs, '5', gprofiler_kwargs={'all_results':True,
 ↪'sources':['GO:BP']})
```

[136]:
```
dpp4_pap = sc.queries.enrich(fbs, '0', org='mmusculus', pval_cutoff=0.05,␣
↪gprofiler_kwargs={'all_results':True})
reticular = sc.queries.enrich(fbs, '1', org='mmusculus', pval_cutoff=0.05,␣
↪gprofiler_kwargs={'all_results':True})
dkk1_pap = sc.queries.enrich(fbs, '3', org='mmusculus', pval_cutoff=0.05,␣
↪gprofiler_kwargs={'all_results':True})
preadipocyte = sc.queries.enrich(fbs, '4', org='mmusculus', pval_cutoff=0.05,␣
↪gprofiler_kwargs={'all_results':True})
div_pap = sc.queries.enrich(fbs, '5', org='mmusculus', pval_cutoff=0.05,␣
↪gprofiler_kwargs={'all_results':True})
```

[137]:
```
dpp4_pap.to_csv('csv/dpp4.csv')
reticular.to_csv('csv/reticular.csv')
dkk1_pap.to_csv('csv/dkk1.csv')
preadipocyte.to_csv('csv/preadipo.csv')
div_pap.to_csv('csv/dividing pap.csv')
```

[79]:
```
fbs.obs['group'] = fbs.obs.cell_type
```

[92]:
```
fbs.obs.group[fbs.obs.leiden.isin(['0','3','5'])] = 'papillary'
```

```
---------------------------------------------------------------------------
TypeError                                 Traceback (most recent call last)
~\OneDrive - Queen Mary, University of␣
↪London\Bioinformatics\bioinf\lib\site-packages\pandas\core\series.py in␣
↪__setitem__(self, key, value)
   1061         try:
-> 1062             self._set_with_engine(key, value)
   1063         except (KeyError, ValueError):

~\OneDrive - Queen Mary, University of␣
↪London\Bioinformatics\bioinf\lib\site-packages\pandas\core\series.py in␣
↪_set_with_engine(self, key, value)
   1094         # fails with AttributeError for IntervalIndex
-> 1095         loc = self.index._engine.get_loc(key)
   1096         # error: Argument 1 to "validate_numeric_casting" has␣
↪incompatible type

~\OneDrive - Queen Mary, University of␣
↪London\Bioinformatics\bioinf\lib\site-packages\pandas\_libs\index.pyx in␣
↪pandas._libs.index.IndexEngine.get_loc()

~\OneDrive - Queen Mary, University of␣
↪London\Bioinformatics\bioinf\lib\site-packages\pandas\_libs\index.pyx in␣
↪pandas._libs.index.IndexEngine.get_loc()

TypeError: 'CellID
P2P21CellCount03:AAGCCGCCACGGCCATx    False
```

```
P2P21CellCount03:AAACGGGTCCAGATCAx     True
P2P21CellCount03:AACTCTTAGCTACCTAx     True
P2P21CellCount03:AAAGATGCATCCTTGCx    False
P2P21CellCount03:AAAGTAGAGGCAGTCAx     True
                                        …
P2P21CellCount05:TTCGAAGGTTCGTGATx    False
P2P21CellCount05:TTCTCAATCCTACAGAx    False
P2P21CellCount05:TTTGGTTTCCCTCTTTx    False
P2P21CellCount05:TTTGCGCTCAGAAATGx     True
P2P21CellCount05:TTGTAGGGTGAAATCAx    False
Name: leiden, Length: 3961, dtype: bool' is an invalid key

During handling of the above exception, another exception occurred:

ValueError                                Traceback (most recent call last)
~\AppData\Local\Temp/ipykernel_9132/640172466.py in <module>
----> 1 fbs.obs.group[fbs.obs.leiden.isin(['0','3','5'])] = 'papillary'

~\OneDrive - Queen Mary, University of␣
↪London\Bioinformatics\bioinf\lib\site-packages\pandas\core\series.py in␣
↪__setitem__(self, key, value)
   1080                 key = np.asarray(key, dtype=bool)
   1081                 try:
-> 1082                     self._where(~key, value, inplace=True)
   1083                 except InvalidIndexError:
   1084                     self.iloc[key] = value

~\OneDrive - Queen Mary, University of␣
↪London\Bioinformatics\bioinf\lib\site-packages\pandas\core\generic.py in␣
↪_where(self, cond, other, inplace, axis, level, errors)
   8857
   8858             self._check_inplace_setting(other)
-> 8859             new_data = self._mgr.putmask(mask=cond, new=other,␣
↪align=align)
   8860             result = self._constructor(new_data)
   8861             return self._update_inplace(result)

~\OneDrive - Queen Mary, University of␣
↪London\Bioinformatics\bioinf\lib\site-packages\pandas\core\internals\managers
↪py in putmask(self, mask, new, align)
    363             new = extract_array(new, extract_numpy=True)
    364
--> 365         return self.apply(

    366             "putmask",
    367             align_keys=align_keys,
```

```
~\OneDrive - Queen Mary, University of␣
↪London\Bioinformatics\bioinf\lib\site-packages\pandas\core\internals\managers
↪py in apply(self, f, align_keys, ignore_failures, **kwargs)
    325                         applied = b.apply(f, **kwargs)
    326                     else:
--> 327                         applied = getattr(b, f)(**kwargs)
    328                 except (TypeError, NotImplementedError):
    329                     if not ignore_failures:

~\OneDrive - Queen Mary, University of␣
↪London\Bioinformatics\bioinf\lib\site-packages\pandas\core\internals\blocks.p␣
↪in putmask(self, mask, new)
   1441                 mask = mask.reshape(new_values.shape)
   1442
-> 1443         new_values[mask] = new
   1444         return [self.make_block(values=new_values)]
   1445

~\OneDrive - Queen Mary, University of␣
↪London\Bioinformatics\bioinf\lib\site-packages\pandas\core\arrays\_mixins.py␣
↪in __setitem__(self, key, value)
    180     def __setitem__(self, key, value):
    181         key = check_array_indexer(self, key)
--> 182         value = self._validate_setitem_value(value)
    183         self._ndarray[key] = value
    184

~\OneDrive - Queen Mary, University of␣
↪London\Bioinformatics\bioinf\lib\site-packages\pandas\core\arrays\categorical
↪py in _validate_setitem_value(self, value)
   2041         # something to np.nan
   2042         if len(to_add) and not isna(to_add).all():
-> 2043             raise ValueError(
   2044                 "Cannot setitem on a Categorical with a new "
   2045                 "category, set the categories first"

ValueError: Cannot setitem on a Categorical with a new category, set the␣
↪categories first
```

[90]:
```
group = fbs.obs.group
```

[100]:
```
fbs.obs.group.cat.add_categories('papillary')
```

[100]:
```
CellID
P2P21CellCount03:AAGCCGCCACGGCCATx           Reticular
P2P21CellCount03:AAACGGGTCCAGATCAx       Dpp4 papillary
P2P21CellCount03:AACTCTTAGCTACCTAx       Dkk1 papillary
```

```
P2P21CellCount03:AAAGATGCATCCTTGCx                      APM
P2P21CellCount03:AAAGTAGAGGCAGTCAx       Dkk1 papillary
                                              …
P2P21CellCount05:TTCGAAGGTTCGTGATx            Reticular
P2P21CellCount05:TTCTCAATCCTACAGAx                  APM
P2P21CellCount05:TTTGGTTTCCCTCTTTx            Reticular
P2P21CellCount05:TTTGCGCTCAGAAATGx    Dividin papillary
P2P21CellCount05:TTGTAGGGTGAAATCAx            Reticular
Name: group, Length: 3961, dtype: category
Categories (10, object): ['APM', 'Adipocyte', 'DP', 'Dividin papillary', …,
'Pericyte', 'Preadipocyte', 'Reticular', 'papillary']
```

[109]:
```
group_label = []
for value in group:
    if 'pap' in value:
        group_label.append('papillary')
    elif 'Ret' in value:
        group_label.append('reticular')
    elif 'Pre' in value:
        group_label.append('reticular')
    else:
        group_label.append('')
```

[111]:
```
fbs.obs.group = group_label
```

[112]:
```
sc.pl.umap(fbs, color='group')
```

```
C:\Users\hhy696\OneDrive - Queen Mary, University of
London\Bioinformatics\bioinf\lib\site-packages\anndata\_core\anndata.py:1220:
FutureWarning: The `inplace` parameter in pandas.Categorical.reorder_categories
is deprecated and will be removed in a future version. Removing unused
categories will always return a new Categorical object.
  c.reorder_categories(natsorted(c.categories), inplace=True)
… storing 'group' as categorical
```

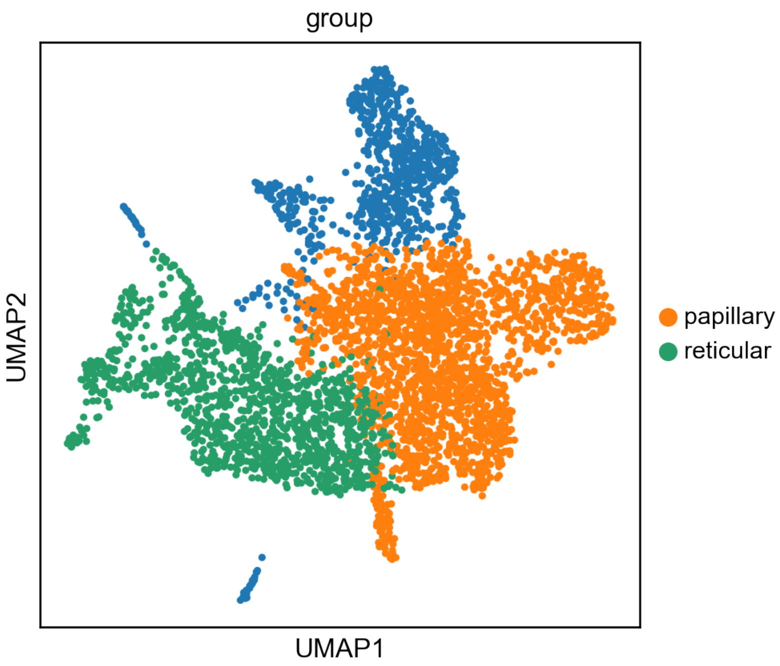

[114]:
```
sc.tl.rank_genes_groups(fbs, 'group', method='t-test',␣
 ↪groups=['papillary','reticular'])
sc.pl.rank_genes_groups(fbs, n_genes=25, sharey=False)
```

```
ranking genes
    finished: added to `.uns['rank_genes_groups']`
    'names', sorted np.recarray to be indexed by group ids
    'scores', sorted np.recarray to be indexed by group ids
    'logfoldchanges', sorted np.recarray to be indexed by group ids
    'pvals', sorted np.recarray to be indexed by group ids
    'pvals_adj', sorted np.recarray to be indexed by group ids (0:00:00)
```

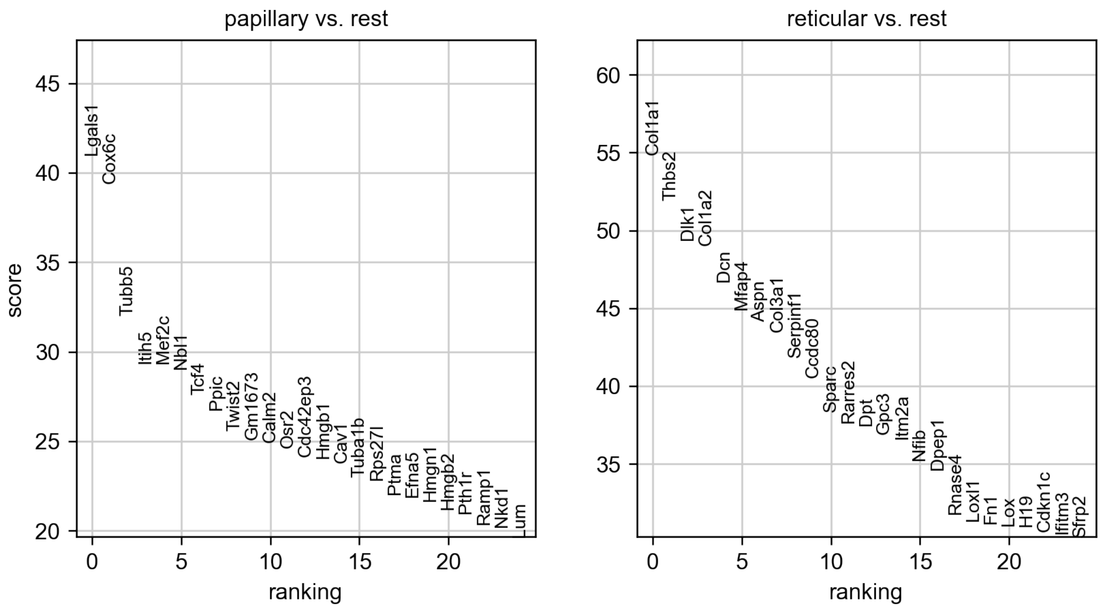

[115]:
```
pap = sc.queries.enrich(fbs, 'papillary', gprofiler_kwargs={'all_results':True,
 ↪'sources':['GO:BP']})
ret = sc.queries.enrich(fbs, 'reticular', gprofiler_kwargs={'all_results':True,
 ↪'sources':['GO:BP']})
```

[116]:
```
pap.to_csv('PvsR.csv')
ret.to_csv('RvsP.csv')
```

[117]:
```
pr = fbs[fbs.obs.group.isin(['papillary','reticular'])]
sc.pl.umap(pr)
```

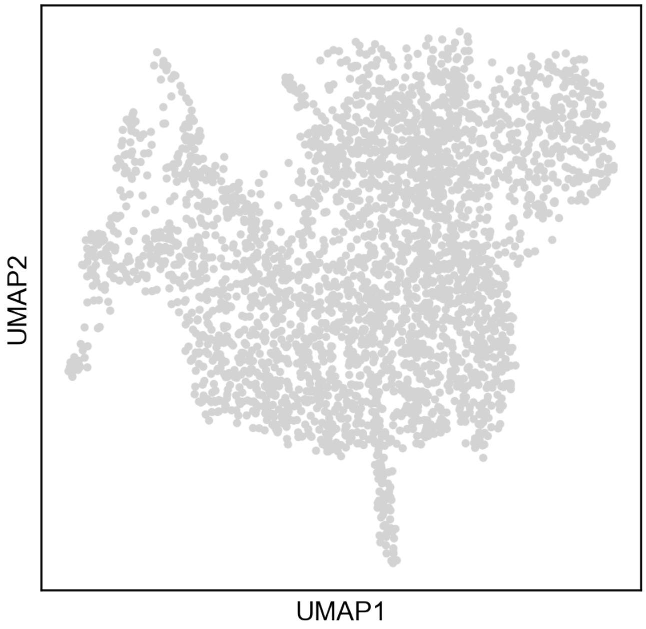


```
[118]: sc.tl.rank_genes_groups(pr, 'group', method='t-test')
       sc.pl.rank_genes_groups(pr, n_genes=25, sharey=False)
```

```
ranking genes

Trying to set attribute `.uns` of view, copying.

    finished: added to `.uns['rank_genes_groups']`
    'names', sorted np.recarray to be indexed by group ids
    'scores', sorted np.recarray to be indexed by group ids
    'logfoldchanges', sorted np.recarray to be indexed by group ids
    'pvals', sorted np.recarray to be indexed by group ids
    'pvals_adj', sorted np.recarray to be indexed by group ids (0:00:00)
```

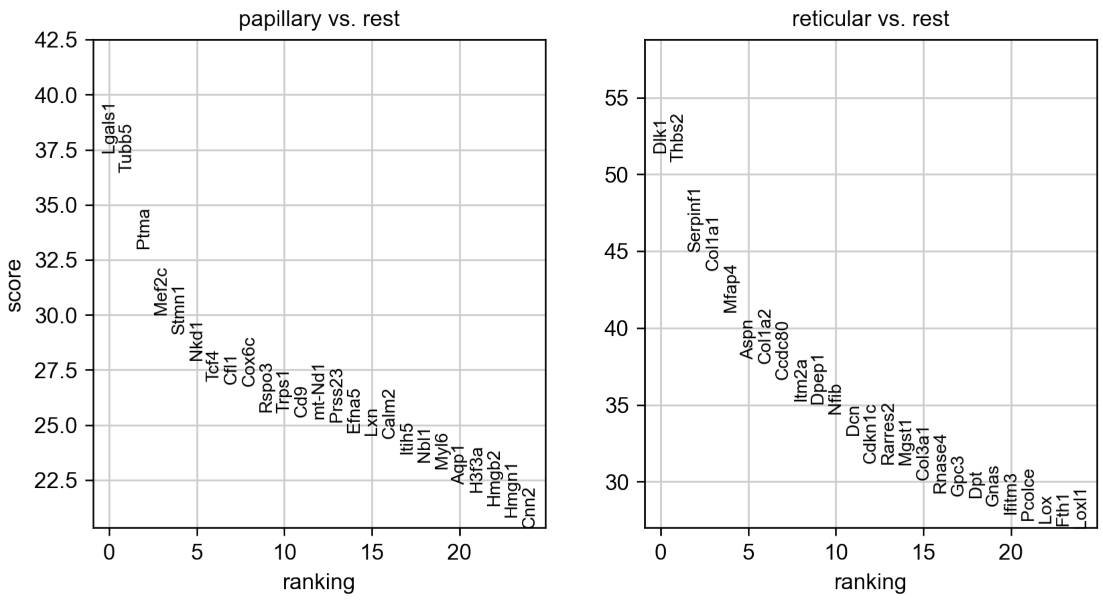

[122]:
```
pap = sc.queries.enrich(pr, 'papillary', org='mmusculus',␣
 ↪gprofiler_kwargs={'all_results':True, 'sources':['GO:BP']})
ret = sc.queries.enrich(pr, 'reticular', org='mmusculus',␣
 ↪gprofiler_kwargs={'all_results':True, 'sources':['GO:BP']})
```

[123]:
```
pap.to_csv('PvsR.csv')
ret.to_csv('RvsP.csv')
```

[121]:
```
sc.queries.enrich(pr, 'papillary', org='mmusculus',␣
 ↪gprofiler_kwargs={'all_results':True, 'sources':['GO:BP']})
```

[121]:
```
       source      native                                         name  \
0       GO:BP  GO:0071840  cellular component organization or biogenesis
1       GO:BP  GO:0044237                    cellular metabolic process
2       GO:BP  GO:0016043               cellular component organization
3       GO:BP  GO:0008152                            metabolic process
4       GO:BP  GO:0006807          nitrogen compound metabolic process
…       …         …                                                  …
12280   GO:BP  GO:0034230                         enkephalin processing
12281   GO:BP  GO:0034238                             macrophage fusion
12282   GO:BP  GO:0034239               regulation of macrophage fusion
12283   GO:BP  GO:0034350     regulation of glial cell apoptotic process
12284   GO:BP  GO:2001311     lysobisphosphatidic acid metabolic process

             p_value  significant  \
0      7.668515e-116         True
1      2.989177e-111         True
```

```
2      1.379987e-105         True
3      2.537420e-100         True
4       1.574006e-88         True
…                …           …
12280   1.000000e+00        False
12281   1.000000e+00        False
12282   1.000000e+00        False
12283   1.000000e+00        False
12284   1.000000e+00        False

                                             description  term_size  \
0      "A process that results in the biosynthesis of…       6270
1      "The chemical reactions and pathways by which …      10350
2      "A process that results in the assembly, arran…       6090
3      "The chemical reactions and pathways, includin…      11429
4      "The chemical reactions and pathways involving…       9669
…                                                  …         …
12280  "The formation of mature enkephalin, a pentape…          3
12281  "The binding and fusion of a macrophage to one…          6
12282  "Any process that modulates the frequency, rat…          5
12283  "Any process that modulates the frequency, rat…         12
12284  "The chemical reactions and pathways involving…          2

       query_size  intersection_size  effective_domain_size  precision  \
0            4480               1976                  21118   0.441071
1            4480               2868                  21118   0.640179
2            4480               1904                  21118   0.425000
3            4480               3057                  21118   0.682366
4            4480               2653                  21118   0.592187
…             …                  …                     …         …
12280        4480                  2                  21118   0.000446
12281        4480                  2                  21118   0.000446
12282        4480                  1                  21118   0.000223
12283        4480                  6                  21118   0.001339
12284        4480                  2                  21118   0.000446

         recall    query                   parents
0      0.315152  query_1              [GO:0009987]
1      0.277101  query_1  [GO:0008152, GO:0009987]
2      0.312644  query_1              [GO:0071840]
3      0.267477  query_1              [GO:0008150]
4      0.274382  query_1              [GO:0008152]
…           …        …                       …
12280  0.666667  query_1              [GO:0016486]
12281  0.333333  query_1              [GO:0000768]
12282  0.200000  query_1  [GO:0034238, GO:0060142]
12283  0.500000  query_1  [GO:0034349, GO:0042981]
```

```
12284  1.000000  query_1  [GO:0006650, GO:0052646]

[12285 rows x 14 columns]
```

[138]:
```
fbs.write('P2 fibroblasts.h5ad', compression='lzf')
```

[151]:
```
UV = pd.read_csv('../Genesets/response to UV go term.csv', header=None)
```

[165]:
```
UV = [x[0] for x in UV.values if x[0] in fbs.raw.var_names]
```

[169]:
```
sc.pl.dotplot(fbs, groupby='cell_type', var_names=UV)
```

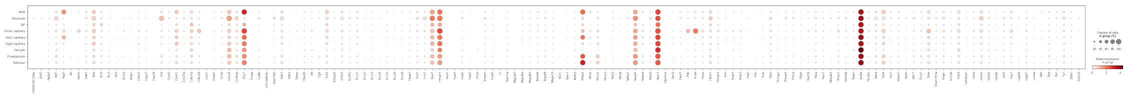

[171]:
```
sc.tl.score_genes(fbs, UV, score_name='UV', use_raw=True)
```

```
computing score 'UV'
    finished: added
    'UV', score of gene set (adata.obs).
    1038 total control genes are used. (0:00:00)
```

[172]:
```
sc.pl.umap(fbs, color='UV')
```

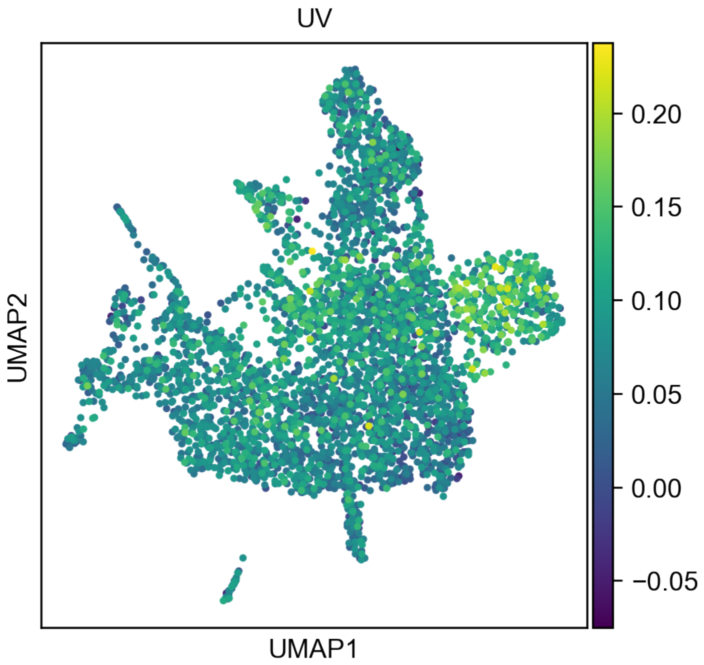

[175]:
```
sc.pl.dotplot(fbs, groupby='cell_type', var_names='UV', standard_scale='var')
```

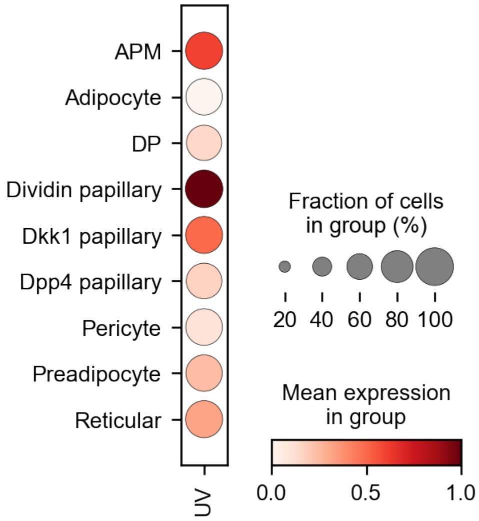

[200]:
```
sc.pl.violin(fbs, groupby='cell_type', keys='UV', rotation=90)
```

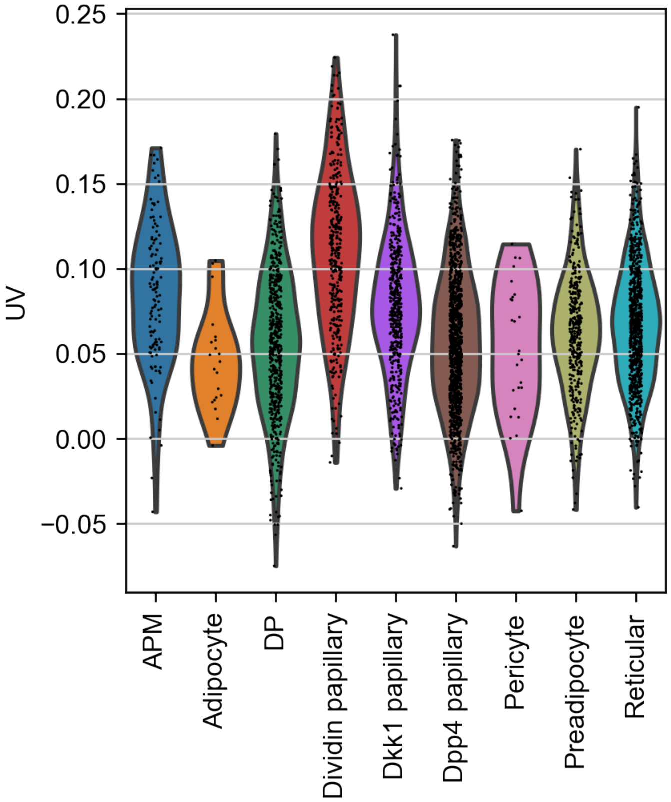

[199]:
```
sc.pl.violin(fbs, groupby='group', keys='UV', standard_scale='var', rotation=90)
```

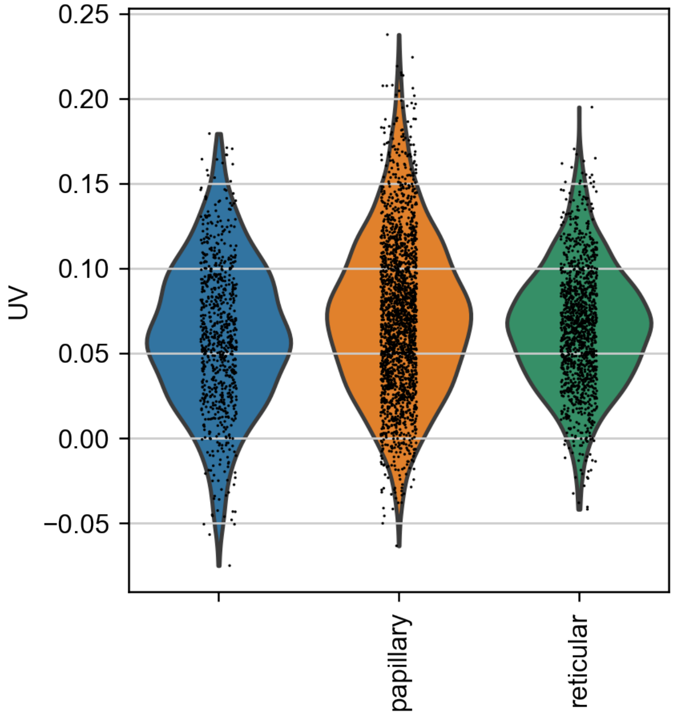

[181]:
```
sc.pl.stacked_violin(fbs, groupby='cell_type', var_names='UV',␣
↪standard_scale='var', rotation=90)
```

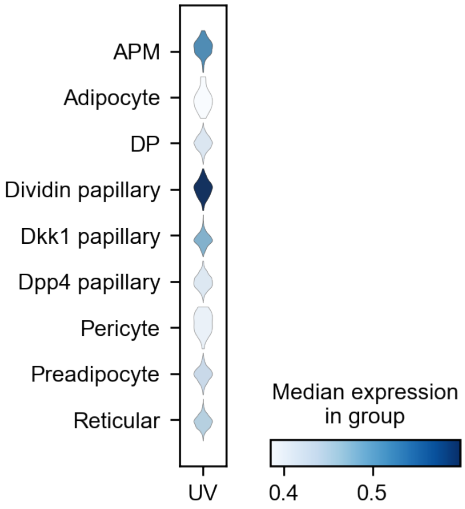

[182]:

```
sc.pl.tracksplot(fbs, groupby='cell_type', var_names='UV',␣
↪standard_scale='var', rotation=90)
```

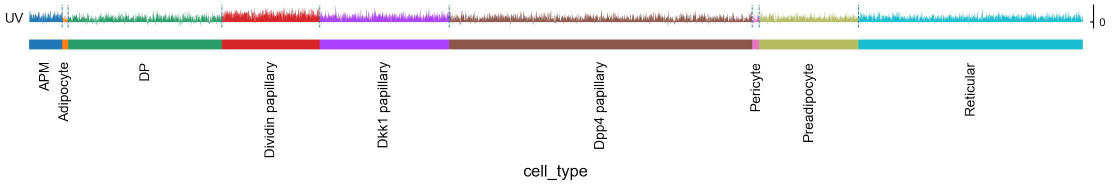

[186]:

```
score = sc.get.obs_df(fbs, keys=['UV', 'cell_type'],)
```

[187]:

```
score
```

[187]:

```
                                          UV        cell_type
CellID
P2P21CellCount03:AAGCCGCCACGGCCATx  0.084893        Reticular
P2P21CellCount03:AAACGGGTCCAGATCAx  0.050989    Dpp4 papillary
P2P21CellCount03:AACTCTTAGCTACCTAx  0.082482    Dkk1 papillary
P2P21CellCount03:AAAGATGCATCCTTGCx  0.104169              APM
P2P21CellCount03:AAAGTAGAGGCAGTCAx  0.027938    Dkk1 papillary
…                                        …                …
P2P21CellCount05:TTCGAAGGTTCGTGATx  0.077811        Reticular
P2P21CellCount05:TTCTCAATCCTACAGAx  0.166840              APM
P2P21CellCount05:TTTGGTTTCCCTCTTTx  0.034200        Reticular
P2P21CellCount05:TTTGCGCTCAGAAATGx  0.082082 Dividin papillary
P2P21CellCount05:TTGTAGGGTGAAATCAx  0.062377        Reticular

[3961 rows x 2 columns]
```

[194]:

```
chart = sns.catplot(x="cell_type", y="UV", kind="box", data=score)
chart.set_xticklabels(rotation=90)
```

[194]:

```
<seaborn.axisgrid.FacetGrid at 0x24e361f2eb0>
```

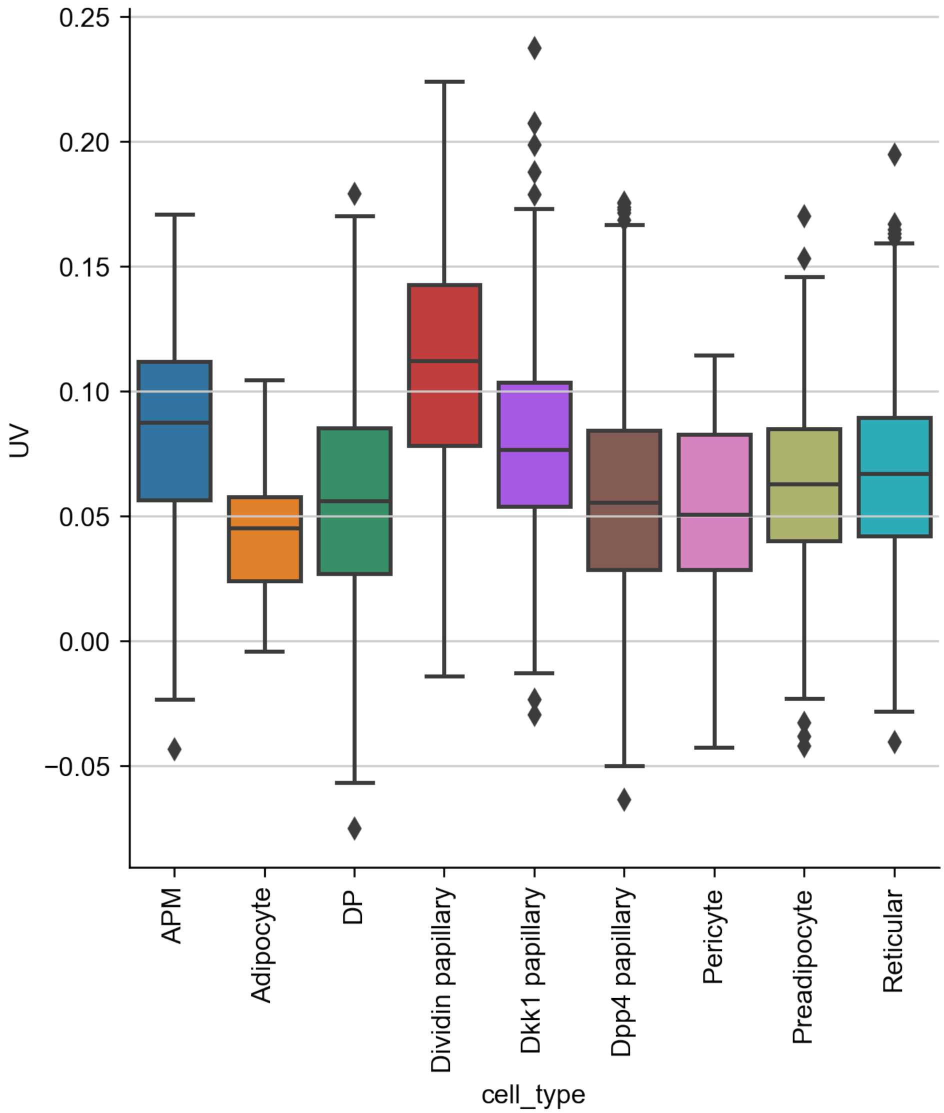

[195]:
```
score2 = sc.get.obs_df(fbs, keys=['UV', 'group'],)
```

[198]:
```
chart = sns.catplot(x="group", y="UV", kind="box", data=score2)
chart.set_xticklabels(rotation=90)
```

[198]: <seaborn.axisgrid.FacetGrid at 0x24e39112760>

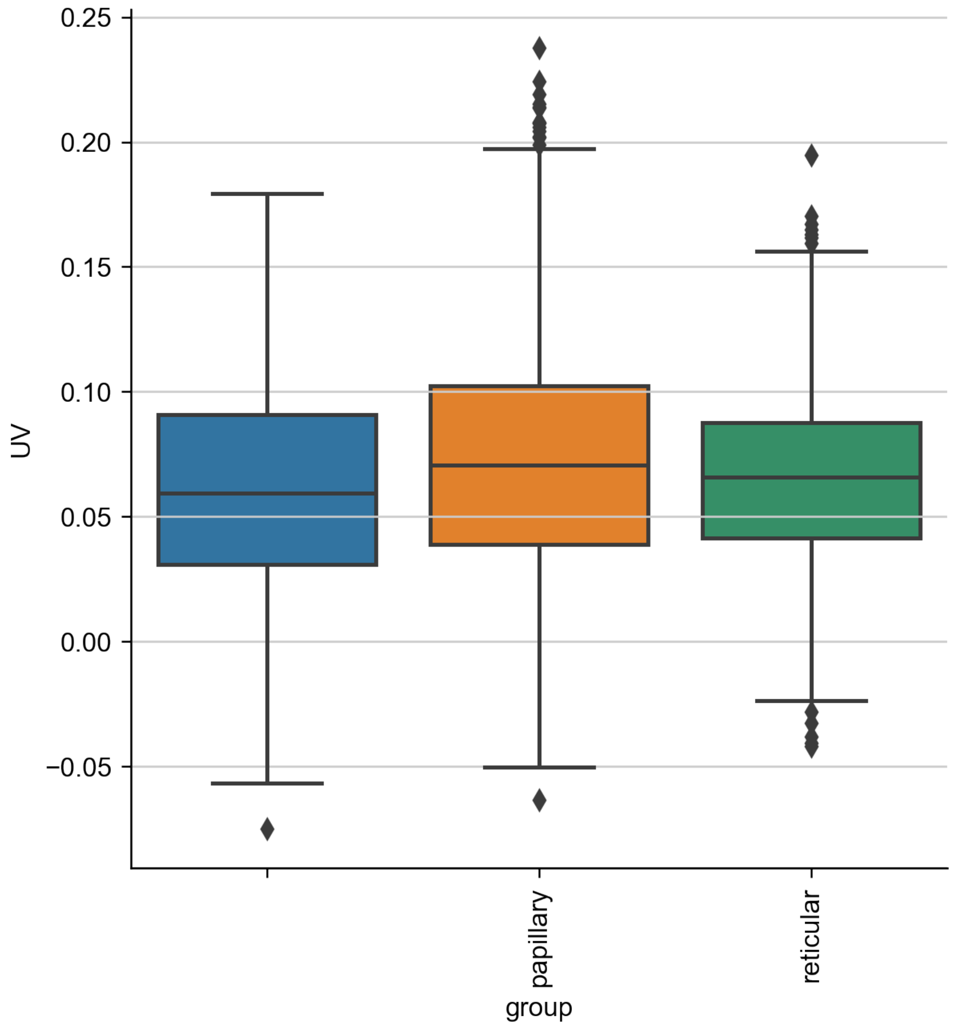

[203]:
```
[print(x, end=', ') for x in UV]
```

```
1700007K13Rik, Actr5, Agap3, Akt1, Aqp1, Atr, Aurkb, Bak1, Bax, Bcl2, Bcl3, Bmf,
Brca2, Brsk1, Casp3, Casp7, Casp9, Cat, Ccar2, Ccnd1, Cdc25a, Cdkn1a, Cdkn2d,
Cers1, Chek1, Cirbp, Cops9, Crebbp, Crip1, Cryaa, Cul4b, D7Ertd443e, Dcun1d3,
Ddb1, Ddb2, Ddias, Dhx36, Dtl, Egfr, Ei24, Eif2ak4, Eif2s1, Ep300, Ercc1, Ercc2,
Ercc3, Ercc4, Ercc5, Ercc6, Ercc8, Fbxw7, Fech, Fmr1, Gpx1, Hmgn1, Hus1, Hyal1,
Hyal2, Hyal3, Il12a, Impact, Ino80, Ivl, Kdm1a, Map2k7, Map3k4, Map4k3, Mapk8,
Mapk9, Mapk13, Mc1r, Men1, Mettl3, Mfap4, Mme, Mmp2, Mmp3, Msh2, Msh6, N4bp1,
Nedd4, Nfatc4, Noc2l, Npm1, Opn1sw, Opn3, Parp1, Pbk, Pclaf, Pcna, Pik3r1,
Pmaip1, Pml, Pold1, Pold3, Polh, Poli, Polk, Ppid, Primpol, Prkaa1, Prkcd,
Ptprk, Rad18, Rela, Rev1, Rhbdd1, Rhno1, Rnf168, Rpain, Rpl26, Ruvbl2, Sde2,
Sdf4, Sirt1, Smpd1, Sprtn, Stk11, Timp1, Tipin, Tmem161a, Triap1, Trim32, Trp53,
Trp53inp1, Uaca, Ube2a, Ube2b, Ube4b, Usf1, Usp1, Usp28, Usp47, Uvssa, Wrn, Xpa,
Xpc, Yy1, Zbtb1, Zranb3,
```

```
[203]: [None,
 None,
 None,
 None,
 None,
 None,
 None,
 None,
 None,
 None,
 None,
 None,
 None,
 None,
 None,
 None,
 None,
 None,
 None,
 None,
 None,
 None,
 None,
 None,
 None,
 None,
 None,
 None,
 None,
 None,
 None,
 None,
 None,
 None,
 None,
 None,
 None,
 None,
 None,
 None,
 None,
 None,
 None,
 None,
 None,
 None,
 None,
```

```
None,
None,
None,
None,
None,
None,
None,
None,
None,
None,
None,
None,
None,
None,
None,
None,
None,
None,
None,
None,
None,
None,
None,
None,
None,
None,
None,
None,
None,
None,
None,
None,
None,
None,
None,
None,
None,
None,
None,
None,
None,
None,
None,
None,
None,
None,
None,
```

```
None,
None,
None,
None,
None,
None,
None,
None,
None,
None,
None,
None,
None,
None,
None,
None,
None,
None,
None,
None,
None,
None,
None,
None,
None,
None,
None,
None,
None,
None,
None,
None,
None,
None,
None,
None,
None,
None,
None,
None,
None,
None,
None,
None,
None,
None]
```

[214]:
```python
score[score.cell_type=='Dpp4 papillary'].UV.mean()
score[score.cell_type=='Reticular'].UV.mean()
score[score.cell_type=='Dpp4 papillary'].UV.std()
score[score.cell_type=='Reticular'].UV.std()
score[score.cell_type=='Dpp4 papillary']
score[score.cell_type=='Reticular']
```

[214]:
```
                                        UV  cell_type
CellID
P2P21CellCount03:AAGCCGCCACGGCCATx  0.084893  Reticular
P2P21CellCount03:AACGTTGCATCGGAAGx  0.037578  Reticular
P2P21CellCount03:AAACCTGAGCAATCTCx  0.055654  Reticular
P2P21CellCount03:AACGTTGTCGGTGTCGx  0.022926  Reticular
P2P21CellCount03:AAGGAGCAGCGTCTATx  0.120349  Reticular
…                                        …          …
P2P21CellCount05:TTTCCTCCATGTTGACx -0.019489  Reticular
P2P21CellCount05:TTGTAGGGTAAGGATTx  0.097729  Reticular
P2P21CellCount05:TTCGAAGGTTCGTGATx  0.077811  Reticular
P2P21CellCount05:TTTGGTTTCCCTCTTTx  0.034200  Reticular
P2P21CellCount05:TTGTAGGGTGAAATCAx  0.062377  Reticular

[844 rows x 2 columns]
```

[215]:
```python
chart = sns.catplot(y="cell_type", x="UV", kind="box", data=score)
chart.set_xticklabels(rotation=90)
```

[215]:
```
<seaborn.axisgrid.FacetGrid at 0x24e476d1f70>
```

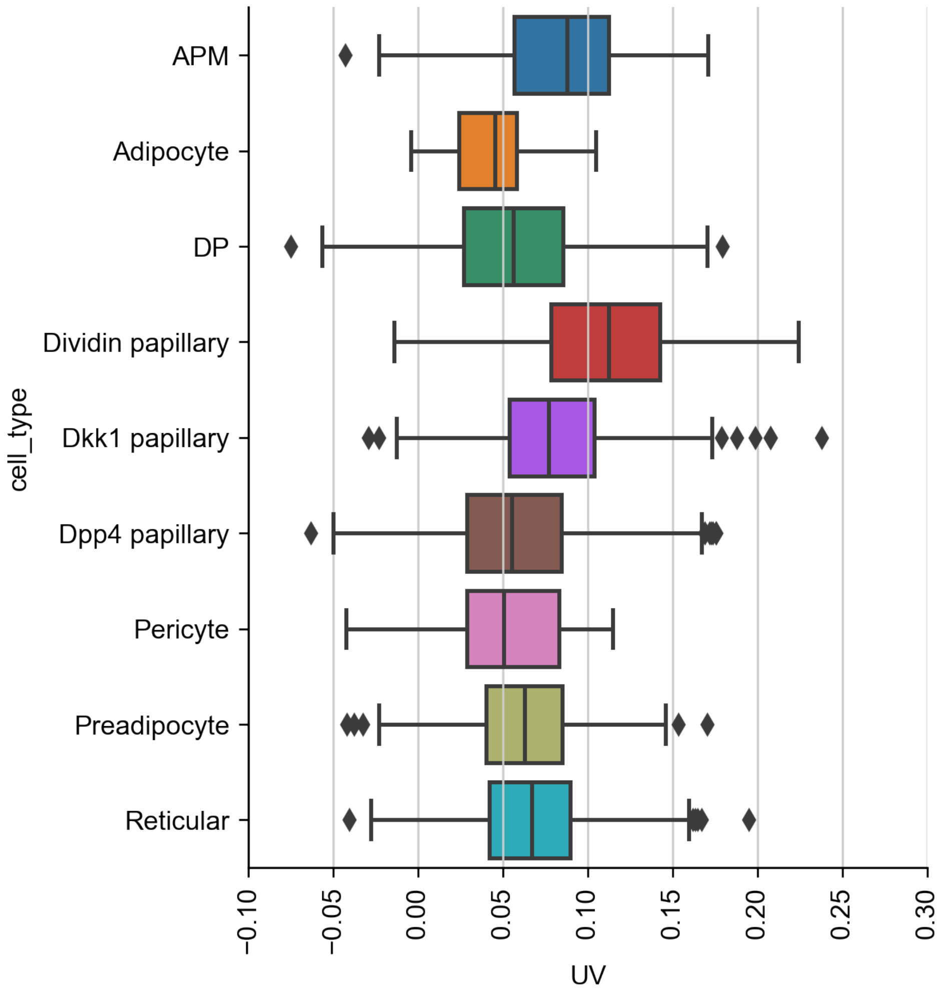

[218]:
```
main_clusters =␣
↪['Fibroblast','Keratinocyte','Fibroblast','Keratinocyte','Fibroblast','Keratinocyte','Fibro
                 'Endothelial','SMC/
↪pericyte','Schwann','Keratinocyte','Melanocyte','Immune','Endothelial','Keratinocyte']
adata.obs['cell_type'] = [main_clusters[int(x)] for x in adata.obs.leiden]
```

[219]:
```
sc.pl.umap(adata, color='cell_type')
```

```
C:\Users\hhy696\OneDrive - Queen Mary, University of
London\Bioinformatics\bioinf\lib\site-packages\anndata\_core\anndata.py:1220:
FutureWarning: The `inplace` parameter in pandas.Categorical.reorder_categories
is deprecated and will be removed in a future version. Removing unused
categories will always return a new Categorical object.
  c.reorder_categories(natsorted(c.categories), inplace=True)
… storing 'cell_type' as categorical
```

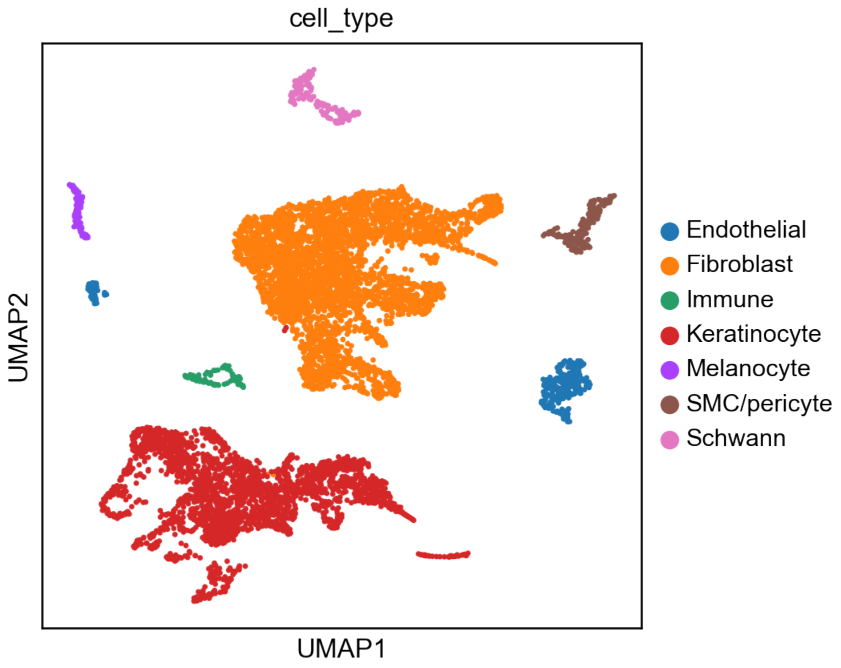


```
[220]: sc.pl.umap(fbs, color='leiden')
```

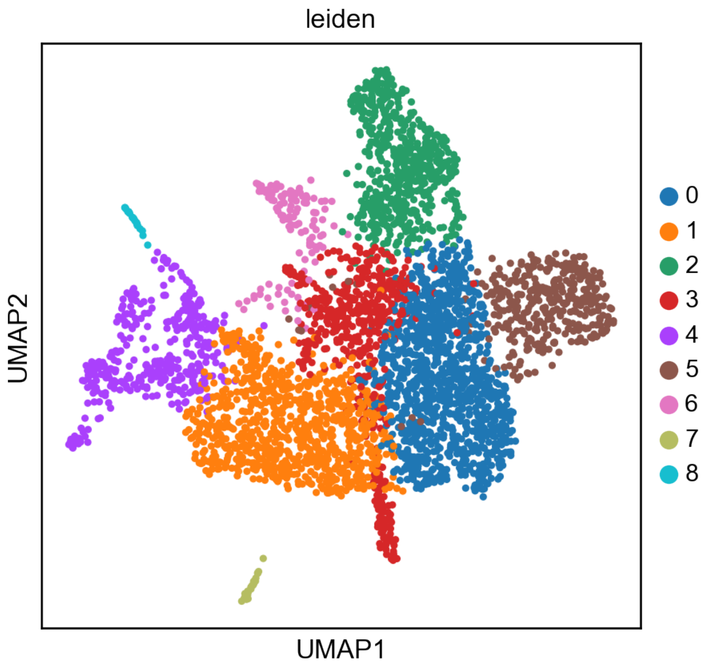

[221]:
```
fb_clusters =␣
↪['Papillary','Reticular','Other','Papillary','Preadipocyte','Papillary','Other','Other','Ad[illegible]
fbs.obs['subtype'] = [fb_clusters[int(x)] for x in fbs.obs.leiden]
```

[226]:
```
sc.pl.umap(fbs, color='subtype',␣
↪groups=['Papillary','Reticular','Preadipocyte','Adipocyte','Other'])
```

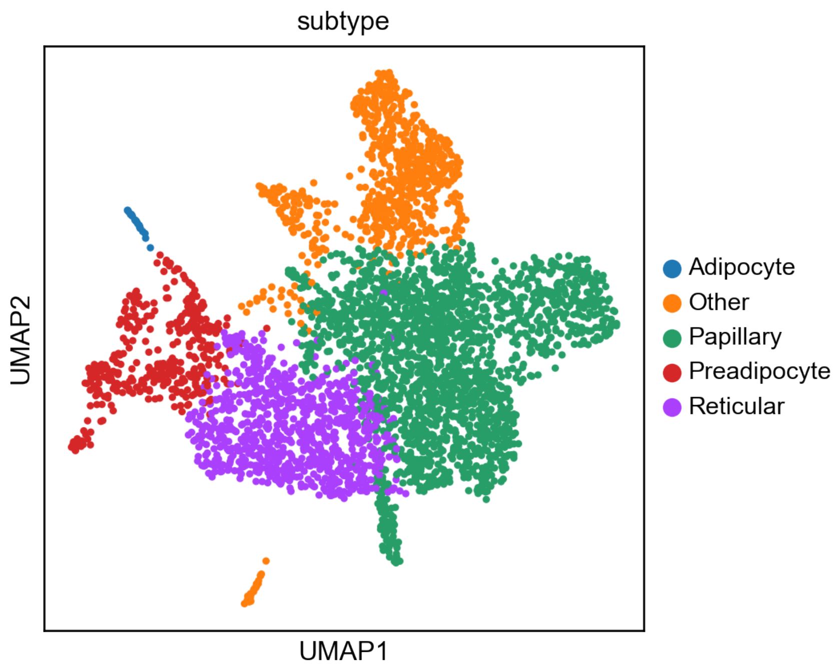

[237]:
```
fbs.obs['subtype'] = fbs.obs['subtype'].cat.
↪reorder_categories(['Papillary','Reticular','Preadipocyte','Adipocyte','Other'])
```

[238]:
```
sc.pl.umap(fbs, color='subtype')
```

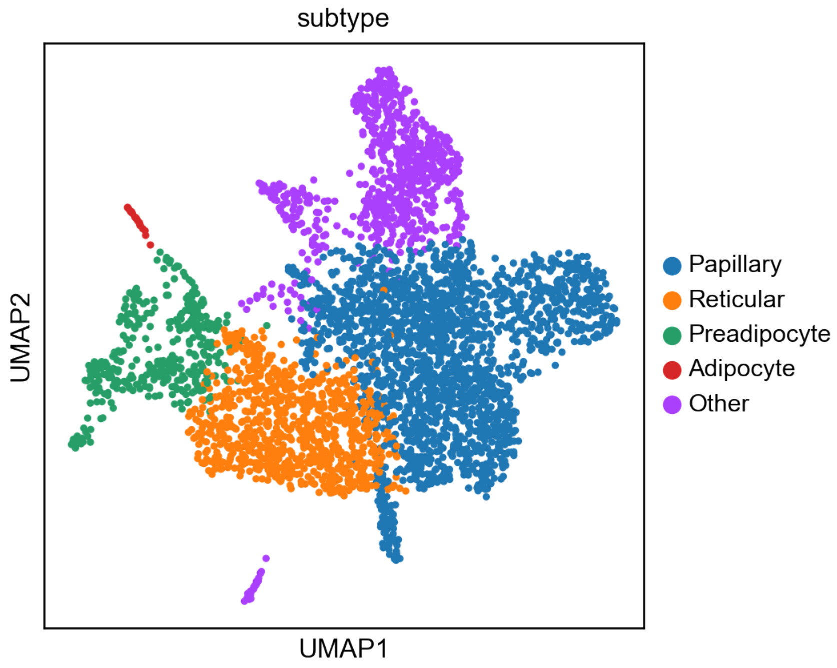

[240]:
```
sc.tl.rank_genes_groups(fbs, 'subtype', method='t-test')
sc.pl.rank_genes_groups(fbs, n_genes=25, sharey=False)
```

```
ranking genes
    finished: added to `.uns['rank_genes_groups']`
    'names', sorted np.recarray to be indexed by group ids
    'scores', sorted np.recarray to be indexed by group ids
    'logfoldchanges', sorted np.recarray to be indexed by group ids
    'pvals', sorted np.recarray to be indexed by group ids
    'pvals_adj', sorted np.recarray to be indexed by group ids (0:00:00)
```

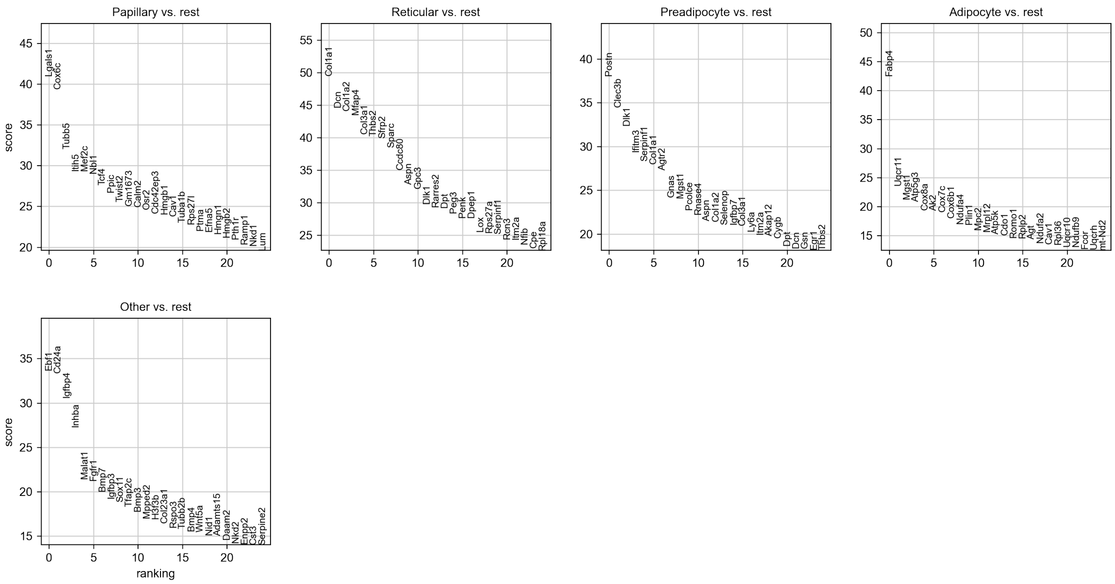

[242]:
```python
pd.DataFrame(fbs.uns['rank_genes_groups']['names']).head(10)
```

[242]:
```
  Papillary Reticular Preadipocyte Adipocyte   Other
0    Lgals1    Col1a1        Postn     Fabp4    Ebf1
1     Cox6c       Dcn       Clec3b    Uqcr11   Cd24a
2     Tubb5    Col1a2         Dlk1     Mgst1  Igfbp4
3     Itih5     Mfap4       Ifitm3    Atp5g3   Inhba
4     Mef2c    Col3a1     Serpinf1     Cox8a  Malat1
5      Nbl1     Thbs2       Col1a1       Ak2   Fgfr1
6      Tcf4     Sfrp2        Agtr2     Cox7c    Bmp7
7      Ppic     Sparc         Gnas    Cox6b1  Igfbp3
8    Twist2    Ccdc80        Mgst1    Ndufa4   Sox11
9    Gm1673      Aspn       Pcolce     Plin1  Tfap2c
```

[247]:
```python
sig_genes = sc.get.rank_genes_groups_df(fbs, group=None, pval_cutoff=0.05,␣
↪log2fc_min=0)
```

[253]:
```python
list(sig_genes[sig_genes.group=='Papillary'].names.values)
```

[253]:
```
['Lgals1',
 'Cox6c',
 'Tubb5',
 'Itih5',
 'Mef2c',
 'Nbl1',
 'Tcf4',
 'Ppic',
 'Twist2',
```

```
'Gm1673',
'Calm2',
'Osr2',
'Cdc42ep3',
'Hmgb1',
'Cav1',
'Tuba1b',
'Rps27l',
'Ptma',
'Efna5',
'Hmgn1',
'Hmgb2',
'Pth1r',
'Ramp1',
'Nkd1',
'Lum',
'Myl6',
'H2afv',
'Rgs10',
'Birc5',
'Hmgn2',
'Pclaf',
'Stmn1',
'H3f3a',
'Ppia',
'Col7a1',
'Wls',
'Maf',
'H2afz',
'Txn1',
'Ran',
'Pitx2',
'Emp2',
'mt-Nd1',
'Itga4',
'Hmgb3',
'Tpm4',
'Prss23',
'Wisp1',
'Top2a',
'Csrp1',
'Cdca3',
'Grem2',
'Tmsb10',
'Lhfp',
'Srsf3',
'Cd9',
```

```
'Htra1',
'Ccnb2',
'Fxyd6',
'Nusap1',
'Dkk1',
'Spc24',
'Stxbp6',
'Cdca8',
'Ccdc42',
'Cdk1',
'Cenpm',
'Mxd3',
'AC154509.1',
'Trps1',
'Lockd',
'Ybx1',
'Cfl1',
'Ccna2',
'Efnb2',
'Pbk',
'Smc2',
'Ranbp1',
'Nucks1',
'Racgap1',
'Nrn1',
'Cenpa',
'Anxa5',
'Adamts5',
'Cnbp',
'Aqp1',
'Mki67',
'Tk1',
'Fam198b',
'Eif5a',
'Tmem9',
'Tyms',
'Prc1',
'Smc4',
'Emp3',
'Lmna',
'Tead2',
'Dnajc9',
'Cenpf',
'Tpx2',
'Anxa2',
'2310022B05Rik',
'Ccnd1',
```

```
'Actb',
'Hmcn1',
'Anp32a',
'Tpst1',
'Atpif1',
'Rpl7',
'Tmem132c',
'Elob',
'Pmf1',
'Ckap2l',
'Eif2s2',
'Eif4a1',
'Tmpo',
'Pfn1',
'Pdap1',
'Rad51ap1',
'Ube2e3',
'Snrpd1',
'Enho',
'Timm13',
'Ska1',
'Vgll3',
'Raly',
'Bex3',
'Cks2',
'Tpm2',
'Phldb2',
'Igsf10',
'Tubb6',
'Lsm4',
'Ptk7',
'Fgfr4',
'Dek',
'Ccnb1',
'Cdc20',
'Slc25a5',
'Cdk4',
'Hmmr',
'Mns1',
'Vim',
'Emid1',
'Cdkn3',
'Mapre2',
'Cenpe',
'Atp5k',
'Megf6',
'Sh3bgrl3',
```

```
'Minos1',
'Asf1b',
'Lig1',
'Psmb6',
'Dkk2',
'Atp5e',
'Emp1',
'Fam174b',
'Pianp',
'Ppp1ca',
'Psma7',
'Rgs2',
'Tmem109',
'Eif1b',
'Nme1',
'Meox2',
'Aurkb',
'Crip1',
'Hmgn3',
'Pimreg',
'Robo2',
'Nat8l',
'Col6a2',
'Ube2c',
'Cnn2',
'Tceal9',
'Lxn',
'Dbf4',
'Gmnn',
'Grem1',
'Incenp',
'Col26a1',
'Sept7',
'Gm14964',
'Ptpa',
'Lsm5',
'Anp32e',
'Cnn3',
'Eef1d',
'Sgo2a',
'Rrm2',
'mt-Nd4',
'Calm1',
'Hnrnpm',
'Dclk1',
'Gltp',
'Ddx39b',
```

```
'Snrpd2',
'Fez1',
'Creb5',
'Ddah1',
'Vdac1',
'Erh',
'4930579G24Rik',
'Atp5h',
'Cdkn2c',
'Syce2',
'Tubb4b',
'Limch1',
'Mbd3',
'Hnrnpa3',
'Nt5e',
'Mad2l1',
'Sgo1',
'Psmc5',
'Melk',
'Sostdc1',
'Ckap2',
'Psma4',
'Aurka',
'Rnaseh2b',
'Entpd1',
'Atp5j2',
'Atp5g1',
'Ywhah',
'Ndc80',
'Axin2',
'Mafb',
'Smdt1',
'Cav2',
'H2afy2',
'Tcf19',
'Casp3',
'Clspn',
'Hnrnpdl',
'H2afx',
'Bub1b',
'Npm1',
'Kif20a',
'Slc25a4',
'Sppl2b',
'Tipin',
'Egfl6',
'Cox7a2',
```

'Cenpp',
'Psmb7',
'Mrfap1',
'B4galnt1',
'Hpf1',
'Nasp',
'Arpp19',
'Chodl',
'Clic1',
'Ppil1',
'Oaz1',
'Ndufa2',
'Atp5o-1',
'Kifc1',
'Hist1h1b',
'mt-Nd5',
'Tpi1',
'Atp5c1',
'Plxna4',
'Glipr2',
'Crabp2',
'Uqcr10',
'Snrpf',
'Diaph3',
'Tmsb4x',
'Cox6a1',
'Cenpq',
'Chmp2b',
'Prdx2',
'Ptges3',
'Psmb3',
'Dynll1',
'Snrpa',
'Spc25',
'Cenpn',
'Vstm2b',
'Fam111a',
'Dhx32',
'Knl1',
'Neil3',
'Kif22',
'Pgrmc1',
'Cdc25c',
'Ostc',
'Nrm',
'Rfc4',
'Ncapg',

```
'Mcm3',
'Slitrk6',
'Bag1',
'Hint1',
'Rpl22l1',
'Nuf2',
'Rbx1',
'Rfc2',
'Ywhae',
'Nudc',
'Prdx1',
'Hirip3',
'Rgs16',
'Shcbp1',
'Cdca5',
'Rps12',
'Akr1a1',
'Rnaseh2c',
'Eif1ax',
'Idh2',
'Mcm5',
'Ccdc124',
'Lmcd1',
'Limd2',
'Lmnb1',
'Plk1',
'Impa2',
'Arhgdia',
'Rrm1',
'Kif2c',
'Hspg2',
'Ncl',
'Ndufa11',
'Eny2',
'Uhrf1',
'Fam107b',
'Cavin3',
'Kif4',
'Anxa6',
'2900026A02Rik',
'Apela',
'Fhod3',
'Calr',
'Ilf2',
'Tacc3',
'Lyar',
'Tmem47',
```

```
'Fus',
'Ubl5',
'Nudt21',
'Rangap1',
'Srsf7',
'Bub1',
'Lmnb2',
'Ewsr1',
'Ndufb8',
'Prdx6',
'Sf3b6',
'Psmc3ip',
'Tacc2',
'Banf1',
'Hnrnpk',
'Ilk',
'Hnrnpab',
'Sap30',
'Depdc1a',
'Ufsp2',
'Uqcrb',
'Mrgprf',
'Sytl2',
'Sapcd2',
'Ncapd2',
'C330027C09Rik',
'a',
'4930444P10Rik',
'Nudcd2',
'Mdh1',
'Ppih',
'Snap23',
'Cep135',
'Uqcrc1',
'Cbx5',
'Kif23',
'Ywhaq',
'Ppp1r1a',
'Slc25a3',
'Rpl7a',
'Ncam1',
'Mtss1',
'Arhgap11a',
'Snrpe',
'Kif11',
'Hist1h2ae',
'Morf4l2',
```

```
'Ndufb6',
'Cox6b1',
'Lsm6',
'Gm48960',
'mt-Nd2',
'Pfdn6',
'Mrpl58',
'Ass1',
'Cox7b',
'Gtf2a2',
'Cux1',
'Fubp1',
'Cfl2',
'Kif20b',
'Mcm6',
'Mcm2',
'Smco3',
'Sost',
'1110004F10Rik',
'Fip1l1',
'Ncaph',
'Sf3b5',
'Esco2',
'Adamts17',
'Nsg1',
'Gas2l3',
'Ptbp1',
'Cdc42',
'Thy1',
'Cenph',
'S100a11',
'Eef1g',
'Lage3',
'Bub3',
'Chaf1b',
'Mis18bp1',
'Gdi2',
'Sdhaf2',
'Ect2',
'Dut',
'Mmd',
'Mettl9',
'Ggh',
'Flnc',
'Fbxo5',
'Anp32b',
'Trir',
```

```
'A430005L14Rik',
'Gapdh',
'Srsf1',
'Prim1',
'Lsm3',
'Rfc5',
'Taldo1',
'Psmc3',
'Arf5',
'Cox8a',
'Snrnp25',
'Pcna',
'Ddah2',
'Rap2b',
'Kif15',
'Dctpp1',
'Snai2',
'Sec61g',
'Zfp608',
'Ezh2',
'Frrs1l',
'Lsm2',
'Oxct1',
'Cox20',
'Dlgap5',
'Hoxd9',
'Hnrnpc',
'Phf5a',
'Tpm3',
'Psma6',
'Map1b',
'Ftl1',
'Chtop',
'Ssb',
'Htr1b',
'Hells',
'Cyp26b1',
'Arhgef7',
'Ola1',
'Slc25a24',
'Ndufa4',
'Tpm1',
'Prelid1',
'Gins2',
'Mrpl42',
'Cenpw',
'Ssrp1',
```

'Rhoa',
'Anln',
'Msrb2',
'G3bp1',
'Bccip',
'Sapcd1',
'Chchd1',
'Sarnp',
'Psrc1',
'Trim59',
'Ndufb7',
'Gpc6',
'Snrpa1',
'Lsm8',
'Dynlrb1',
'Smad1',
'Mrps14',
'Psma2',
'C530008M17Rik',
'Crabp1',
'Xylt1',
'Cep55',
'Cdca2',
'Dgcr6',
'Prmt1',
'Rgma',
'Pa2g4',
'Vax2os',
'Kpna2',
'Palld',
'Ap2s1',
'Ube2t',
'Cdc25b',
'Lrrc10b',
'Nap1l1',
'Dynlt1f',
'Dpy30',
'Emilin3',
'St8sia2',
'Fbxo17',
'Nhp2',
'Usp1',
'Ndufb10',
'Fbn2',
'Bax',
'Cd34',
'Ap2m1',

```
'Cdc45',
'Pkmyt1',
'Cwc15',
'Ndufs6',
'Ivns1abp',
'Cthrc1',
'Wdr1',
'Tedc1',
'Myof',
'Orc6',
'B3glct',
'Bok',
'Jpt2',
'Cenpx',
'Cfdp1',
'Dkk3',
'Psmc4',
'Nt5dc2',
'Sdhb',
'Tomm22',
'Uqcrq',
'Prpf19',
'Rfc3',
'Ndufaf2',
'Fkbp2',
'Psma1',
'Mrpl17',
'Hnrnpd',
'Pin1',
'Tfdp1',
'Ndufa1',
'Psmd1',
'Chd3os',
'Prss35',
'Eif3h',
'Pid1',
'Eri1',
'Atp5j',
'Dscam',
'Gpx8',
'Cdca4',
'Reep5',
'Hist1h1e',
'Cnnm1',
'Ubl4a',
'Timm50',
'Haus1',
```

```
'Spsb2',
'Sf3b2',
'Smc6',
'Dscc1',
'Mgst3',
'Atp5f1',
'4930511E03Rik',
'Psma3',
'Rbm8a',
'Fkbp1a',
'Hnrnpu',
'Mme',
'Shisa2',
'Prim2',
'Psat1',
'Psmd8',
'Lrr1',
'Trim36',
'Atp6v1g1',
'Ckap5',
'Haus8',
'Vrk1',
'Ash2l',
'Cenpu',
'Mrpl18',
'Tsn',
'Trip13',
'Tmtc2',
'Ddx39',
'1700025G04Rik',
'Lrrfip2',
'Dusp3',
'Nav1',
'Tubg1',
'Slain1',
'Slc48a1',
'Bud31',
'Cops6',
'Hprt',
'Nek2',
'Ch25h',
'Rpa2',
'Impdh2',
'Papola',
'Uqcc2',
'Mrpl28',
'Fabp5',
```

```
'Mthfd2',
'Wif1',
'Arl6ip1',
'Smim5',
'Vps36',
'Fh1',
'Znhit1',
'Hist1h3c',
'Amotl2',
'Cmtm7',
'Phgdh',
'Pomp',
'Map4',
'Cald1',
'Dnajc8',
'Snx8',
'Cdh11',
'Gtdc1',
'Itpa',
'Col8a1',
'Pgm5',
'Tcf12',
'Exosc8',
'Gja1',
'Lrig1',
'Tbcb',
'Anapc5',
'Samm50',
'Nsmce2',
'Cntn4',
'Mtus1',
'Pdia5',
'Rwdd1',
'Ddx41',
'Prr11',
'Higd1a',
'Lims1',
'Atp5a1',
'Prdx4',
'Snrpd3',
'Mis18a',
'Hnrnpr',
'Tpr',
'Cycs',
'2010107E04Rik',
'Bean1',
'Rex1bd',
```

'Tomm7',
'Rpl8',
'Anapc11',
'Vax2',
'Ndufb11',
'Cox5b',
'Anapc13',
'Bcl2l12',
'Cox5a',
'Nans',
'Rad21',
'Gusb',
'Calm3',
'Pmaip1',
'Cdkn2d',
'Smc1a',
'Prpf31',
'Ska3',
'Haspin',
'Aspm',
'Chchd2',
'Mycn',
'Abhd6',
'Actl6a',
'Npdc1',
'Ehd4',
'Psmc2',
'Actr3',
'Ackr3',
'E2f8',
'Arhgef39',
'Tiparp',
'Ndufs4',
'Rspo3',
'Snrnp40',
'Mif',
'Pkm',
'Vdac3',
'Rpl13a',
'Pde1b',
'D8Ertd738e',
'Sf3a1',
'Sugt1',
'Arf1',
'Ndufs8',
'Sdc2',
'Hist1h4i',

```
'Nuggc',
'Epc1',
'Tgfbi',
'Eloc',
'Hikeshi',
'Csnk2b',
'R3hdm4',
'Cep89',
'Eif4h',
'Slirp',
'Eif4a3',
'Hjurp',
'Eif1ad',
'Ube2d3',
'Csnk1a1',
'Tagln2',
'Plk4',
'Nudt19',
'Dcakd',
'Selenow',
'Ncapd3',
'1500009L16Rik',
'Smim10l2a',
'Aimp1',
'Hdac1',
'Stard7',
'Atox1',
'Pola2',
'Myh10',
'Atp5b',
'Ttk',
'Uqcr11',
'Galk1',
'Plekha1',
'Impa1',
'Blvra',
'Rabl6',
'Snx7',
'Slc35b1',
'Kcnab2',
'Smarcb1',
'Anxa1',
'Fam126b',
'Cyp27a1',
'Pdlim1',
'Mid1ip1',
'Tpbg',
```

```
'Iqgap3',
'Snf8',
'Filip1',
'Ppp2r2c',
'Clns1a',
'Olfm1',
'Hmgn5',
'Commd10',
'Gtf3c6',
'C1d',
'Asl',
'Nubp1',
'U2af1',
'Atad2',
'Hnrnpf',
'Dtymk',
'Gm17315',
'Cit',
'Mmp16',
'Dnmt1',
'Mrps21',
'Ucn2',
'BC030867',
'Papss1',
'Tagln',
'Arl6ip5',
'Romo1',
'Elovl6',
'Dazap1',
'Siva1',
'Stk25',
'Serp1',
'Rbm3',
'Mrpl20',
'Matr3',
'Thoc7',
'Capzb',
'Acp1',
'Spata13',
'Ssna1',
'Mcm7',
'5033430I15Rik',
'Serbp1',
'Tmed9',
'Arpc2',
'Sephs1',
'Rpa1',
```

```
'Rps2',
'Sorl1',
'Fam83d',
'Mrps25',
'Gmps',
'Mrto4',
'Cplx2',
'Cct3',
'Lypd1',
'E2f1',
'Epn2',
'Mlf1',
'Kifc5b',
'Mcrip1',
'Hsbp1',
'Gm16183',
'Pole3',
'St6galnac3',
'Eme1',
'Rfc1',
'Tmem98',
'Mrpl54',
'Mdk',
'Fen1',
'Commd3',
'Selenof',
'Cenpk',
'Thyn1',
'Dhfr',
'Tgfb1',
'Hnrnph1',
'Ywhab',
'Il13ra1',
'Nde1',
'Swi5',
'Myl12a',
'Cyba',
'Id1',
'Nop10',
'Zranb2',
'P4ha2',
'Tuba1c',
'Ccdc141',
'Mad1l1',
'Lta4h',
'Rps19',
'Wnt16',
```

```
'Hdac2',
'Nup43',
'Set',
'Haus4',
'Psmc1',
'Twist1',
'Cct5',
'Atp5g3',
'Trp53bp2',
'Coro1c',
'Cdh13',
'Zmat5',
'Fam105a',
'Cbx1',
'Mybl2',
'Bora',
'Ankle1',
'Coch',
'Nrtn',
'Lmf2',
'Gas1',
'Tmem123',
'Skp2',
'Cdt1',
'Smc3',
'Sh3bp4',
'Ntf3',
'Fkbp3',
'Psmd6',
'Gsdmd',
'Metap2',
'Cdc6',
'Pold2',
'Gm38037',
'Troap',
'Adgrl2',
'Ppp1r7',
'Sh3rf3',
'Gng2',
'Immp1l',
'Gm10282',
'Snrpb2',
'Smyd2',
'Bzw2',
'Ddx1',
'Cisd1',
'Eif3c',
```

```
'Nsl1',
'E2f7',
'Ccne2',
'Rpp25l',
'Dmd',
'Psmb4',
'Vdac2',
'Aunip',
'Exo1',
'Ndufc1',
'Hmg20b',
'Ndufv3',
'Mis12',
'Poc1a',
'Spdl1',
'Wnt11',
'Uxs1',
'Hnrnph3',
'Cetn2',
'Zwilch',
'Cby1',
'Pold1',
'Me1',
'Bzw1',
'G2e3',
'Rbbp8',
'Elof1',
'Neurl1b',
'Ndufv2',
'Nxph3',
'Mrpl52',
'Trub1',
'Samd4',
'Rab1b',
'Ndufb9',
'Mrps15',
'Espl1',
'Dbi',
'Rheb',
'Tnfrsf12a',
'Pbdc1',
'Gnb1',
'Ppa1',
'Nono',
'Sltm',
'Prkag3',
'Slc7a2',
```

```
'Polr2i',
'Slco3a1',
'Cdr2',
'Uchl5',
'Actr1a',
'C1qtnf2',
'Dpp4',
'Hlf',
'Vps13b',
'Flna',
'U2surp',
'Fdps',
'Ift43',
'Flot1',
'C1qtnf1',
'Nfic',
'Ralb',
'Ppib',
'Mfap2',
'Zfp367',
'Cacybp',
'Fam96a',
'Fam96b',
'Psmd14',
'Opn3',
'Map2k1',
'Enkd1',
'Stil',
'Foxm1',
'Fundc2',
'Lima1',
'Srp14',
'Rab8b',
'Gtse1',
'Tctn3',
'Gde1',
'Elovl4',
'Rpn2',
'Ncaph2',
'Psd3',
'Mrpl51',
'Snrpc',
'Gm17018',
'4930503L19Rik',
'Rcc1',
'Psmd2',
'Ryr3',
```

```
'Sf3a2',
'Mrps11',
'E330013P04Rik',
'Sfxn3',
…]
```

[255]:
```
pap = sc.queries.enrich(list(sig_genes[sig_genes.group=='Papillary'].names.
↪values), org='mmusculus', gprofiler_kwargs={'all_results':True, 'sources':
↪['GO:BP']})
```

[256]:
```
ret = sc.queries.enrich(list(sig_genes[sig_genes.group=='Reticular'].names.
↪values), org='mmusculus', gprofiler_kwargs={'all_results':True, 'sources':
↪['GO:BP']})
preadipo = sc.queries.enrich(list(sig_genes[sig_genes.group=='Preadipocyte'].
↪names.values), org='mmusculus', gprofiler_kwargs={'all_results':True,␣
↪'sources':['GO:BP']})
adipo = sc.queries.enrich(list(sig_genes[sig_genes.group=='Adipocyte'].names.
↪values), org='mmusculus', gprofiler_kwargs={'all_results':True, 'sources':
↪['GO:BP']})
```

[257]:
```
pap.to_csv('csv/pap.csv')
ret.to_csv('csv/ret.csv')
preadipo.to_csv('csv/preadipo.csv')
adipo.to_csv('csv/adipo.csv')
```

[ ]:
```
```
