# Supplementary material for: Role of distinct fibroblast lineages and immune cells in dermal repair following UV radiation-induced tissue damage
Source: eLife. 2021 Dec 23;10:e71052. doi: 10.7554/eLife.71052 (PMC8747514; doi:10.7554/eLife.71052)
Supplement: Figure 1—figure supplement 2—source data 1. — Files include the analysis protocol files of P2 and P21 mouse skin single-cell RNA-seq datasets and GO term enrichment analysis of indicated datasets and figure panel. [file elife-71052-fig1-figsupp2-data1.zip › Figure 1- supplementary Souce file 2/Figure S2C - P21 UV analysis.pdf]

P21 UV analysis

November 19, 2021

[1]:
```
import os
import numpy as np
import pandas as pd
os.environ['PATH'] += os.pathsep + os.path.
 ↪expanduser('~\\AppData\\Roaming\\Python\\Lib\\site-packages\\tables')
import scanpy as sc
import loompy as lp
#from MulticoreTSNE import MulticoreTSNE as TSNE
import json
import base64
import zlib
from pyscenic.plotting import plot_binarization
#from pyscenic.export import add_scenic_metadata
#from pyscenic.cli.utils import load_signatures
import matplotlib as mpl
import matplotlib.pyplot as plt
#from scanpy.plotting._tools.scatterplots import plot_scatter
#import scanpy.pl.scatter as plot_scatter
import seaborn as sns
```

[2]:
```
adata = sc.read( 'all_cells.loom', validate=False)
```

```
Variable names are not unique. To make them unique, call
`.var_names_make_unique`.
```

[3]:
```
adata.var_names_make_unique()
```

[4]:
```
adata = adata[adata.obs.age==21]
```

[5]:
```
# Set up some scanpy preferences
sc.settings.verbosity = 3 # verbosity: errors (0), warnings (1), info (2),␣
 ↪hints (3)
#sc.logging.print_versions()
sc.set_figure_params(dpi=120, fontsize=10, dpi_save=600)

# Set maximum number of jobs for Scanpy. Stops the computer freezing up using␣
 ↪all the cores
sc.settings.njobs = 4
```

[6]:
```
sc.pl.highest_expr_genes(adata, n_top=20, )
```

```
C:\Users\hhy696\OneDrive - Queen Mary, University of
London\Bioinformatics\bioinf\lib\site-
packages\scanpy\preprocessing\_normalization.py:155: UserWarning: Revieved a
view of an AnnData. Making a copy.
  view_to_actual(adata)
```

```
normalizing counts per cell
    finished (0:00:00)
```

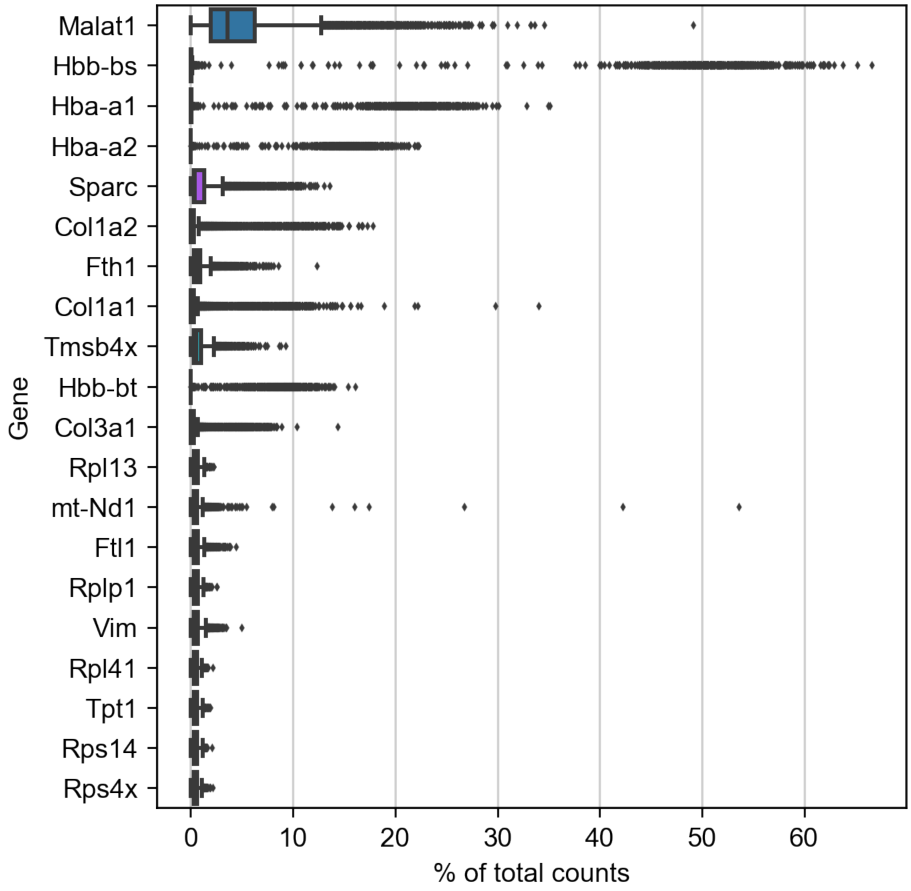

[7]:
```
# filter cells with less than 200 genes expressed or genes expressed in less␣
↪than 3 cells
sc.pp.filter_cells(adata, min_genes=200)
sc.pp.filter_genes(adata, min_cells=3)
```

```
filtered out 447 cells that have less than 200 genes expressed
filtered out 14028 genes that are detected in less than 3 cells
```

[8]:
```
sc.pp.calculate_qc_metrics(adata, percent_top=None, log1p=False, inplace=True)
```

[9]:
```
sc.pl.violin(adata, ['n_genes_by_counts', 'total_counts'],
             jitter=0.4, multi_panel=True)
```

```
C:\Users\hhy696\OneDrive - Queen Mary, University of
London\Bioinformatics\bioinf\lib\site-packages\anndata\_core\anndata.py:1220:
FutureWarning: The `inplace` parameter in pandas.Categorical.reorder_categories
is deprecated and will be removed in a future version. Removing unused
categories will always return a new Categorical object.
  c.reorder_categories(natsorted(c.categories), inplace=True)
… storing 'CreationDate' as categorical
C:\Users\hhy696\OneDrive - Queen Mary, University of
London\Bioinformatics\bioinf\lib\site-packages\anndata\_core\anndata.py:1220:
FutureWarning: The `inplace` parameter in pandas.Categorical.reorder_categories
is deprecated and will be removed in a future version. Removing unused
categories will always return a new Categorical object.
  c.reorder_categories(natsorted(c.categories), inplace=True)
… storing 'last_modified' as categorical
C:\Users\hhy696\OneDrive - Queen Mary, University of
London\Bioinformatics\bioinf\lib\site-packages\anndata\_core\anndata.py:1220:
FutureWarning: The `inplace` parameter in pandas.Categorical.reorder_categories
is deprecated and will be removed in a future version. Removing unused
categories will always return a new Categorical object.
  c.reorder_categories(natsorted(c.categories), inplace=True)
… storing 'Chromosome' as categorical
C:\Users\hhy696\OneDrive - Queen Mary, University of
London\Bioinformatics\bioinf\lib\site-packages\anndata\_core\anndata.py:1220:
FutureWarning: The `inplace` parameter in pandas.Categorical.reorder_categories
is deprecated and will be removed in a future version. Removing unused
categories will always return a new Categorical object.
  c.reorder_categories(natsorted(c.categories), inplace=True)
… storing 'Strand' as categorical
```

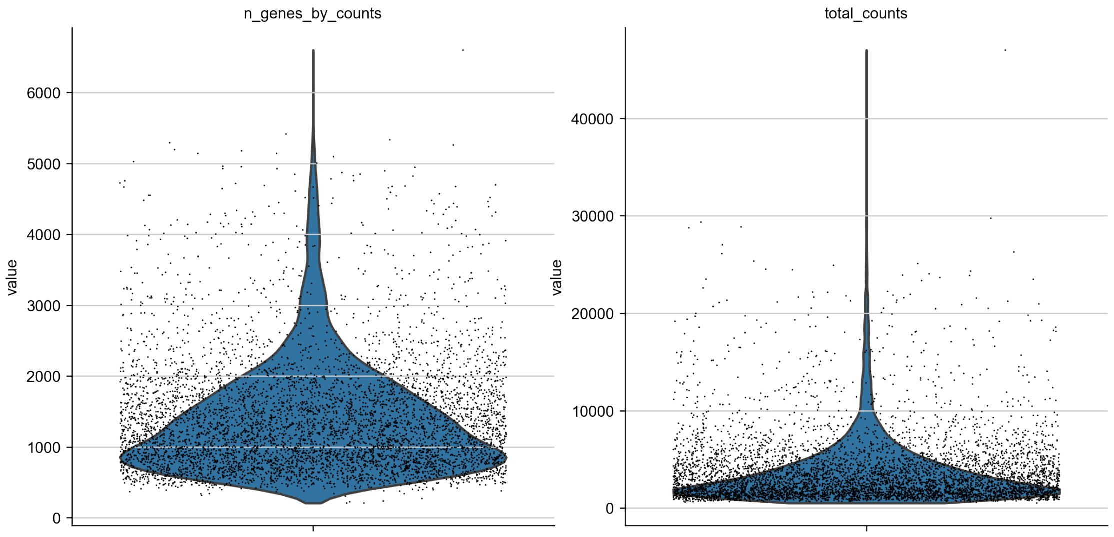

[10]:
```
sc.pl.scatter(adata, x='total_counts', y='n_genes_by_counts')
```

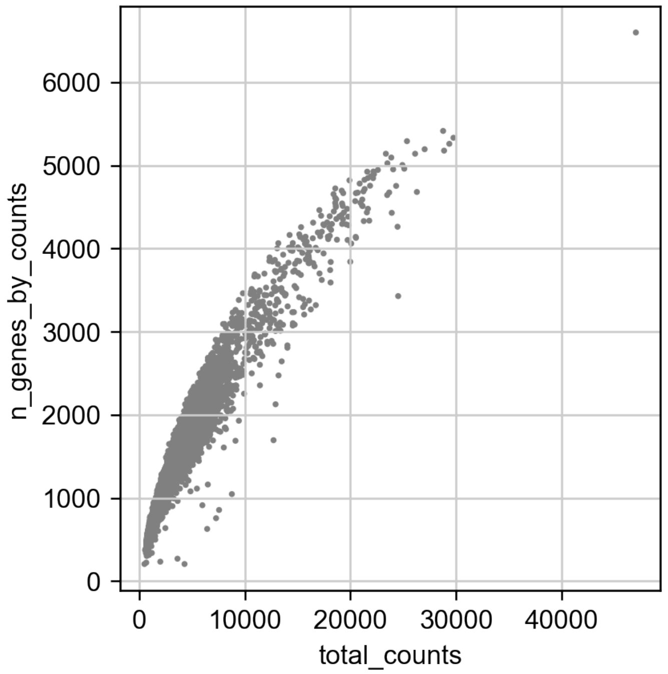

[11]:
```
# Do some more preprocessing https://scanpy-tutorials.readthedocs.io/en/latest/
↪pbmc3k.html
sc.pp.normalize_total(adata, target_sum=1e4)
sc.pp.log1p(adata)
sc.pp.highly_variable_genes(adata, min_mean=0.0125, max_mean=3, min_disp=0.5)
sc.pl.highly_variable_genes(adata)
```

```
normalizing counts per cell
    finished (0:00:00)
extracting highly variable genes
    finished (0:00:00)
--> added
    'highly_variable', boolean vector (adata.var)
    'means', float vector (adata.var)
    'dispersions', float vector (adata.var)
    'dispersions_norm', float vector (adata.var)
```

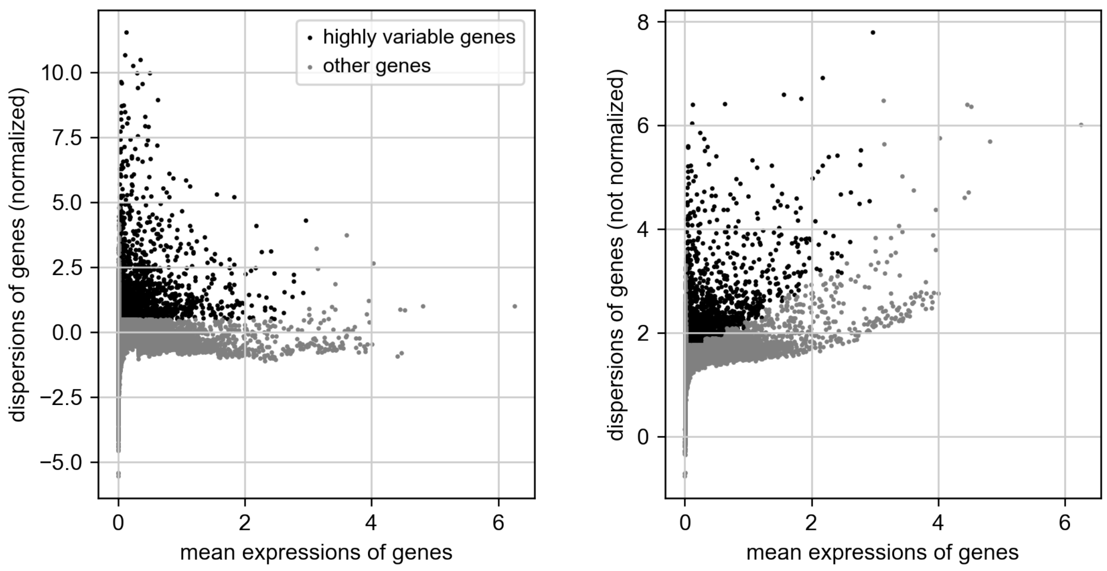

[12]:
```
# save a copy of the raw data. This is sometimes used by other functions later
adata.raw = adata
```

[13]:
```
# more preprocessing
adata = adata[:, adata.var.highly_variable]
sc.pp.regress_out(adata, ['total_counts'])
sc.pp.scale(adata, max_value=10)
```

```
regressing out ['total_counts']
    sparse input is densified and may lead to high memory use
    finished (0:00:14)
```

[14]:
```
# run principal component analysis
sc.tl.pca(adata, n_comps=30)
```

```
computing PCA
    on highly variable genes
    with n_comps=30
    finished (0:00:01)
```

[15]:
```
# plot the PCA, colouring by wounded and some fibroblast genes
sc.pl.pca(adata, color=['age', 'sample', 'Pdgfra', 'Twist2', 'Col1a2'], alpha=0.
 ↪5)
```

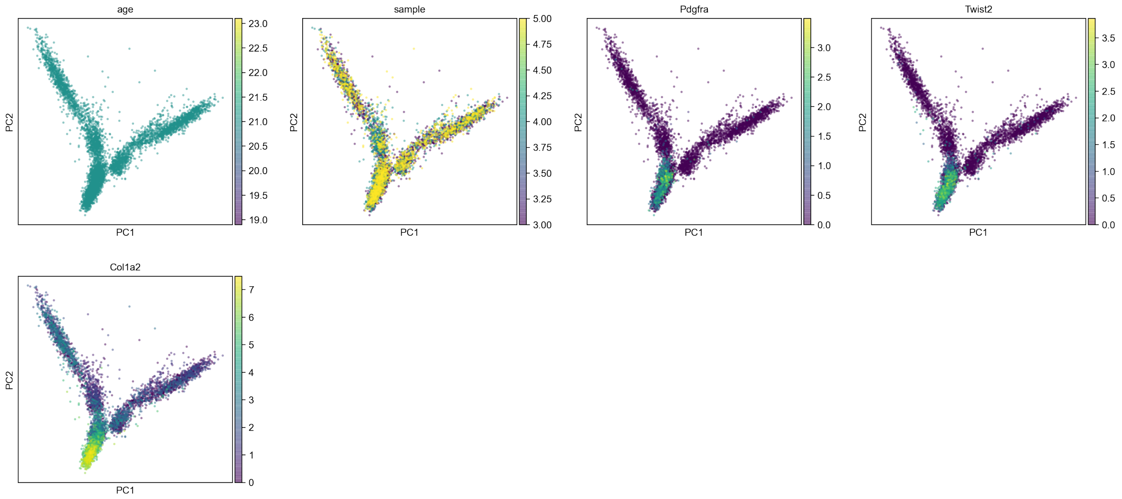


```
[16]: adata.obs['sample'] = adata.obs['sample'].astype('category')
```

```
[17]: # plot the contribution of each PC to the variance
      sc.pl.pca_variance_ratio(adata, log=True)
```

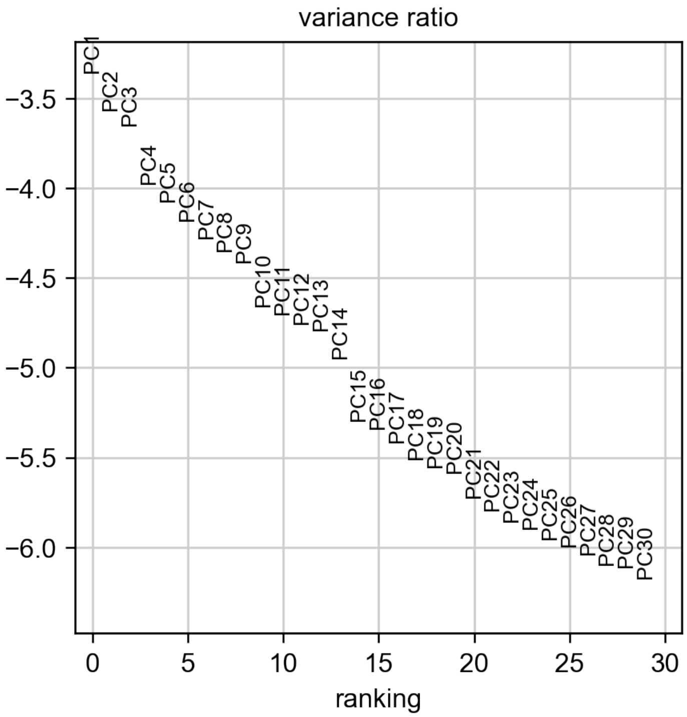

[18]:
```python
# write your filtered dataset
adata.write('P21 cells filtered.h5ad', compression='lzf')
```

[19]:
```python
# compute umap embedding
sc.pp.neighbors(adata, n_neighbors=10)
sc.tl.umap(adata)
sc.pl.umap(adata, color=['sample', 'age', 'Pdgfra', 'Twist2', 'Col1a2'])
```

```
computing neighbors
    using 'X_pca' with n_pcs = 30
    finished: added to `.uns['neighbors']`
    `.obsp['distances']`, distances for each pair of neighbors
    `.obsp['connectivities']`, weighted adjacency matrix (0:00:05)
computing UMAP
    finished: added
    'X_umap', UMAP coordinates (adata.obsm) (0:00:09)
```

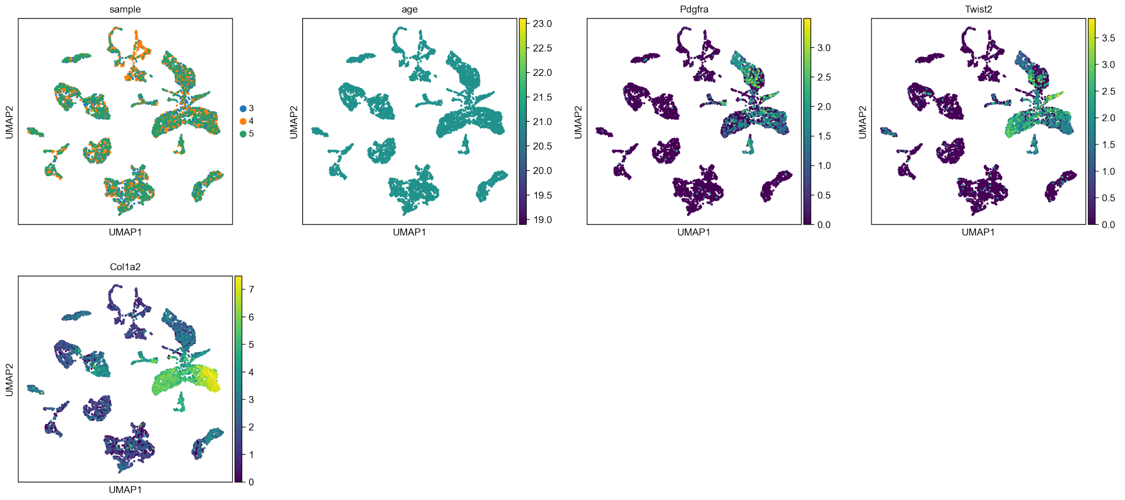

[20]:
```python
sc.pl.umap(adata, color=['sample', 'age', 'Pdgfra', 'Twist2', 'Col1a2'])
```

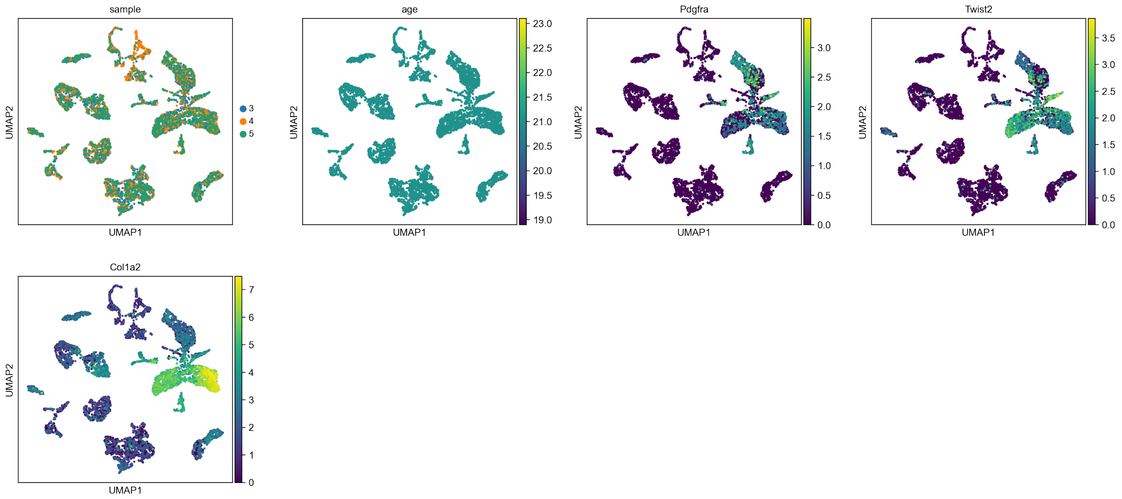

[28]:
```
# cluster the data
sc.tl.leiden(adata, resolution=0.05)
sc.pl.umap(adata, color=['leiden'])
```

```
running Leiden clustering
    finished: found 11 clusters and added
    'leiden', the cluster labels (adata.obs, categorical) (0:00:00)
```

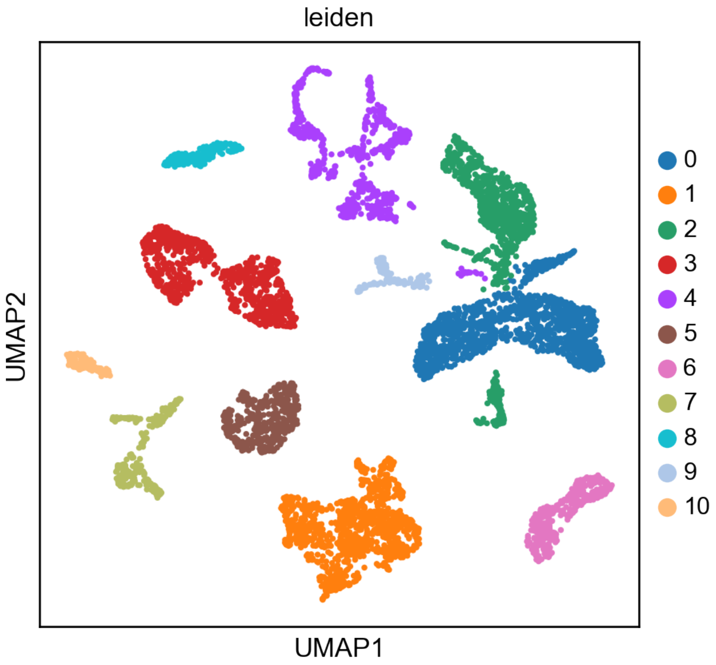

[29]:

```
sc.pl.umap(adata, color=['En1', 'Pdgfra', 'Twist2'] ) # fibroblasts
sc.pl.umap(adata, color=['Col1a1', 'Vim', 'Thy1'] )
sc.pl.umap(adata, color=['Krt10', 'Krt14'] ) # keratinocytes
sc.pl.umap(adata, color=['Pecam1', 'Ly6e', 'Ecscr'] ) # endothelial (Ly6e+) and lymphatic (Ly6e-)
sc.pl.umap(adata, color=['Laptm5', 'Cd53', 'Coro1a'] )# immune cells
sc.pl.umap(adata, color=['Rgs5', 'Mylk'] )              # pericytes
sc.pl.umap(adata, color=['Dct', 'Ptgds'] )              # melanocytes
sc.pl.umap(adata, color=['Plp1', 'Dbi'] )               # Schwann cells (developing)
sc.pl.umap(adata, color=['Etv1', 'Fth1'] )              # Scwann cells (homeostasis)
```

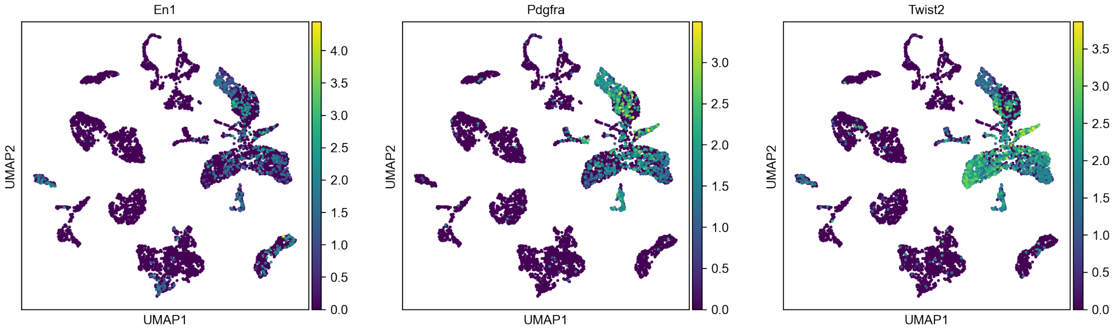

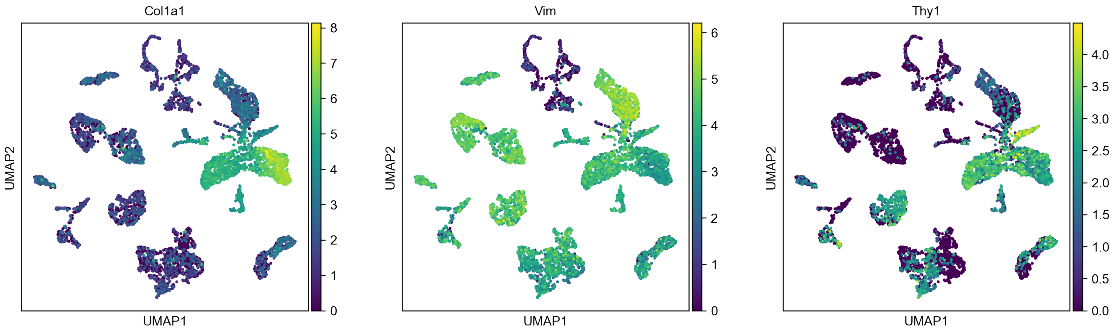

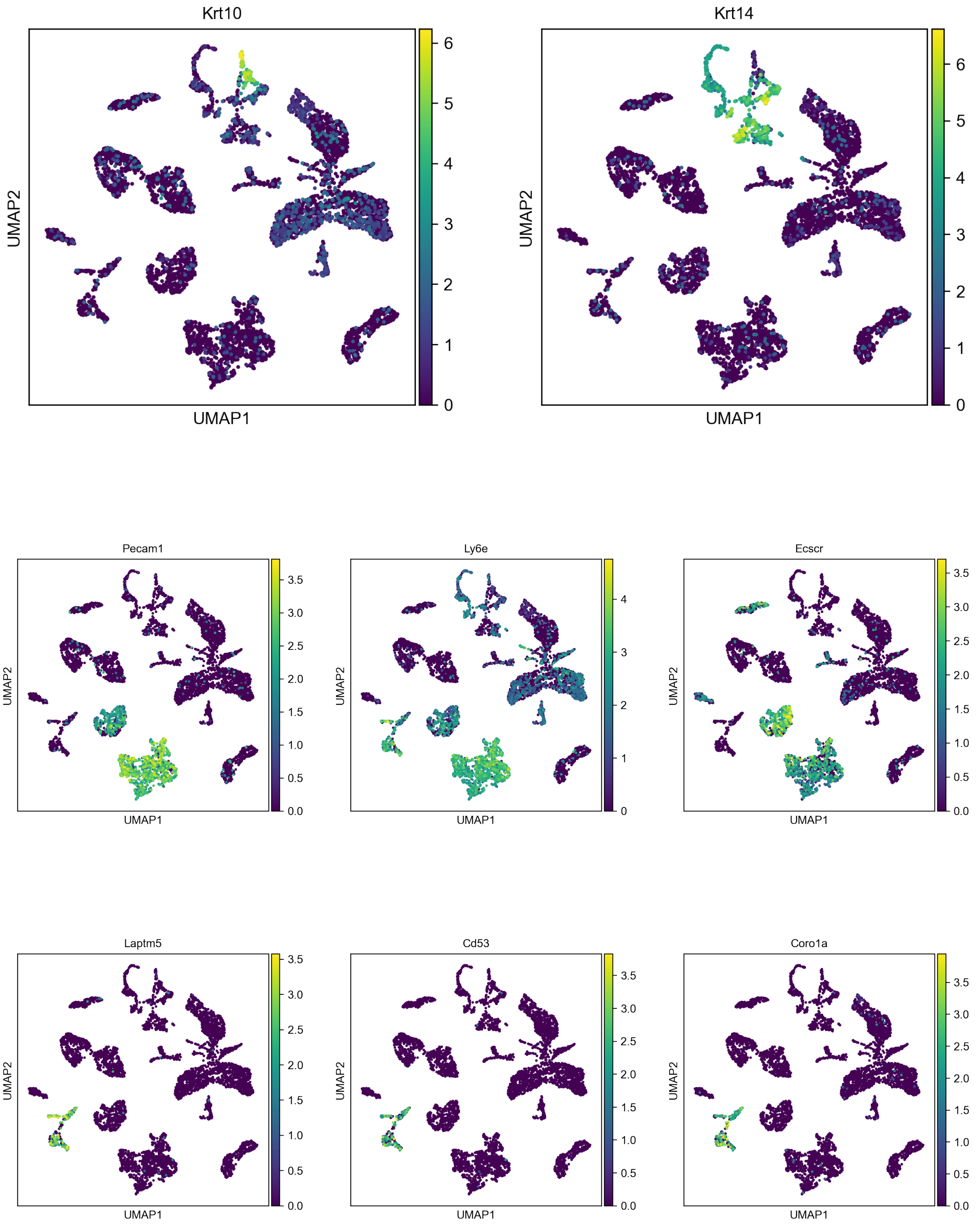

Krt10
Krt14
Pecam1
Ly6e
Ecscr
Laptm5
Cd53
Coro1a
UMAP1
UMAP2

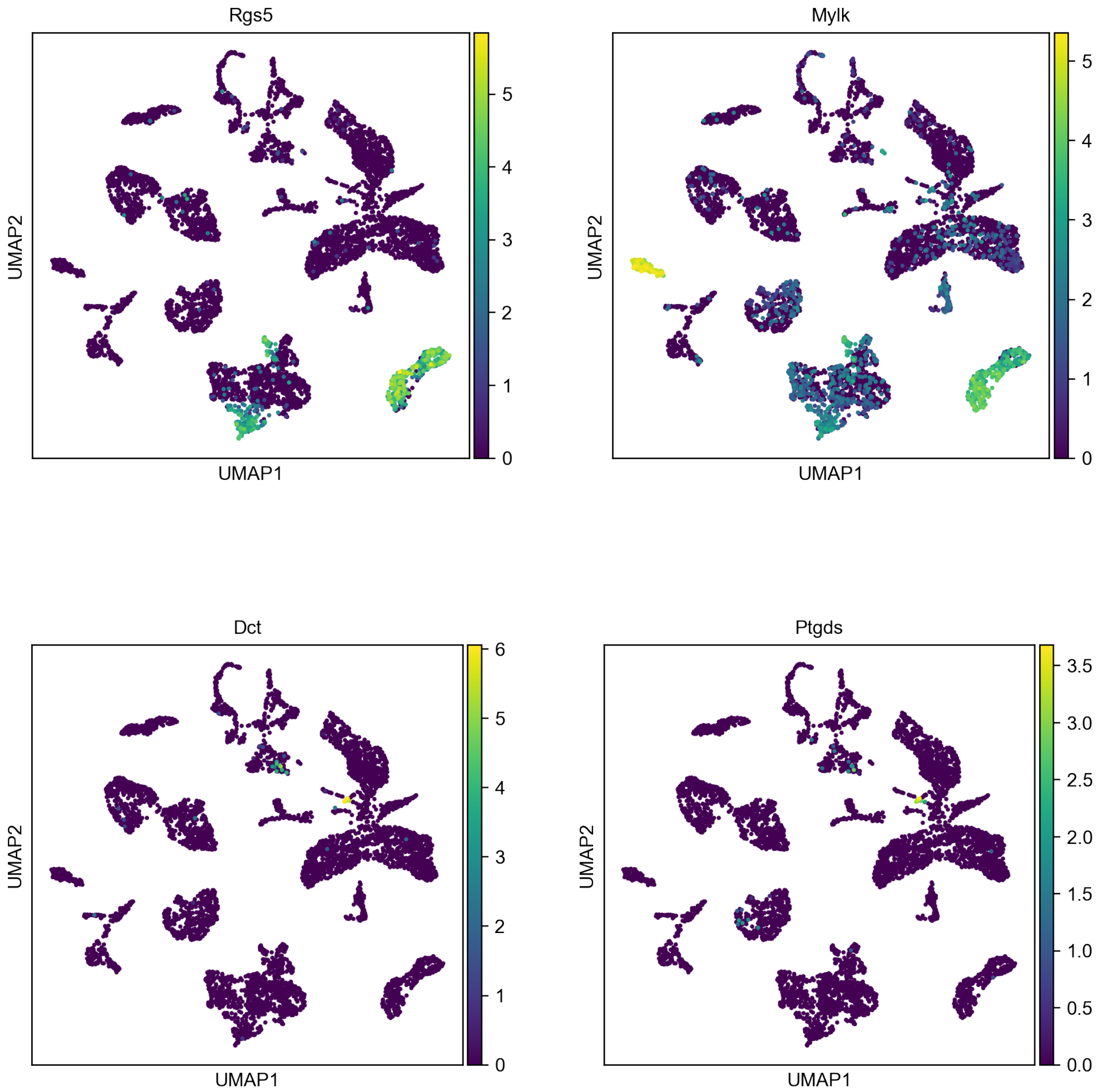

Rgs5
UMAP2
UMAP1
0
1
2
3
4
5
Mylk
UMAP2
UMAP1
0
1
2
3
4
5
Dct
UMAP2
UMAP1
0
1
2
3
4
5
6
Ptgds
UMAP2
UMAP1
0.0
0.5
1.0
1.5
2.0
2.5
3.0
3.5

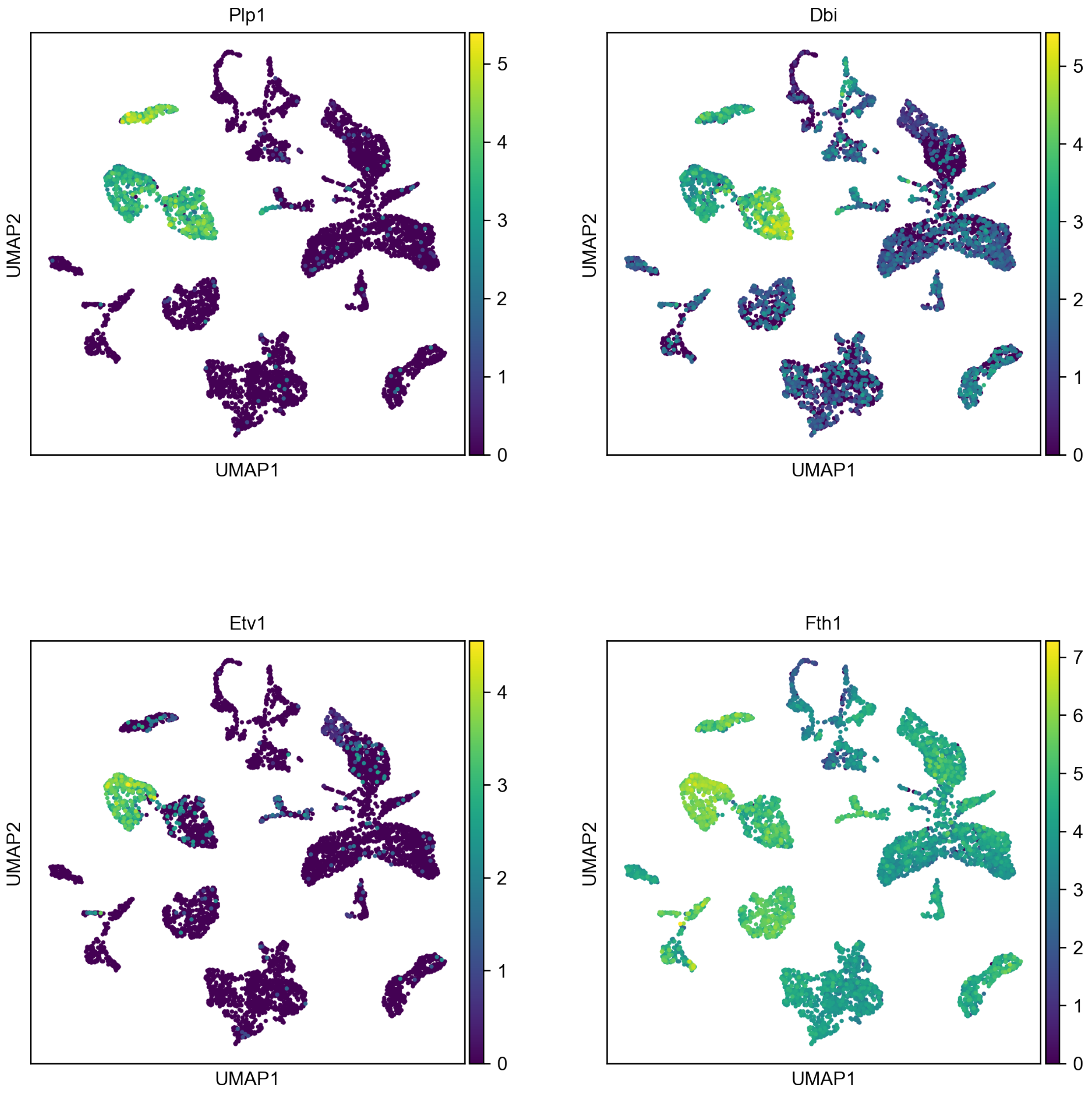

[36]:
```python
sc.tl.rank_genes_groups(adata, 'leiden', method='t-test')
pd.DataFrame(adata.uns['rank_genes_groups']['names']).head(10)
```

```
ranking genes
    finished: added to `.uns['rank_genes_groups']`
    'names', sorted np.recarray to be indexed by group ids
    'scores', sorted np.recarray to be indexed by group ids
    'logfoldchanges', sorted np.recarray to be indexed by group ids
    'pvals', sorted np.recarray to be indexed by group ids
    'pvals_adj', sorted np.recarray to be indexed by group ids (0:00:01)
```

[36]:
```
          0       1       2      3       4      5       6       7  \
0       Dcn    Ly6e  Igfbp3   Plp1  Lgals7  Cldn5    Mylk  Tmsb4x
```

```
1    Col1a1    Pecam1   Malat1     Ptn      Perp    Fxyd6      Rgs5       Fau
2    Col1a2      Cd93   Lgals1     Dbi     Fxyd3    Gng11   Sparcl1  Sh3bgrl3
3     Sparc   Tspan13        a   Timp3     Krt14    Fth1     Crip1      Ucp2
4     Mfap2    Adgrf5    Inhba   Sparc   S100a14    Prox1     Cald1    Laptm5
5  Serpinh1    Ifitm3    Fgfr1   Cd59a    Trim29   Mmrn1      Myl9       B2m
6       Lum      Cdh5      Vim   S100b       Sfn   Ecscr      Tpm1    Coro1a
7      Cd63     Plvap     Bmp4   Gpm6b     Krt15   Pard6g     Tpm2     H2-D1
8     Mfap4    Tm4sf1     Gpx1    Fth1    Spint2    Aplp2    Acta2      Srgn
9     Htra1    Scarb1     Dkk2   Fxyd1     Anxa8     Fgl2       Des   Arhgdib

       8        9      10
0    Mpz      Dcn    Mylk
1    Mbp      Gsn   Acta2
2  Pmp22   Igfbp6   Myh11
3  Ncmap    Mfap5    Tpm2
4  Cryab    Tgfbi    Myl9
5   Gatm     Ebf2    Tpm1
6   Plp1      Mgp     Des
7    Mal   Col3a1   Cald1
8   Pllp   Col1a2   Tagln
9  Kcna1    Cldn1    Myl6
```

[37]:

```python
main_clusters = ['Fibroblast','Endothelial','Fibroblast','Schwann','Keratinocyte','Endothelial','Pericyte',
                 'Immune','Schwann','Fibroblast','SMC']
adata.obs['cell_type'] = [main_clusters[int(x)] for x in adata.obs.leiden]
sc.pl.umap(adata, color='cell_type')
```

```
C:\Users\hhy696\OneDrive - Queen Mary, University of
London\Bioinformatics\bioinf\lib\site-packages\anndata\_core\anndata.py:1220:
FutureWarning: The `inplace` parameter in pandas.Categorical.reorder_categories
is deprecated and will be removed in a future version. Removing unused
categories will always return a new Categorical object.
  c.reorder_categories(natsorted(c.categories), inplace=True)
… storing 'cell_type' as categorical
```

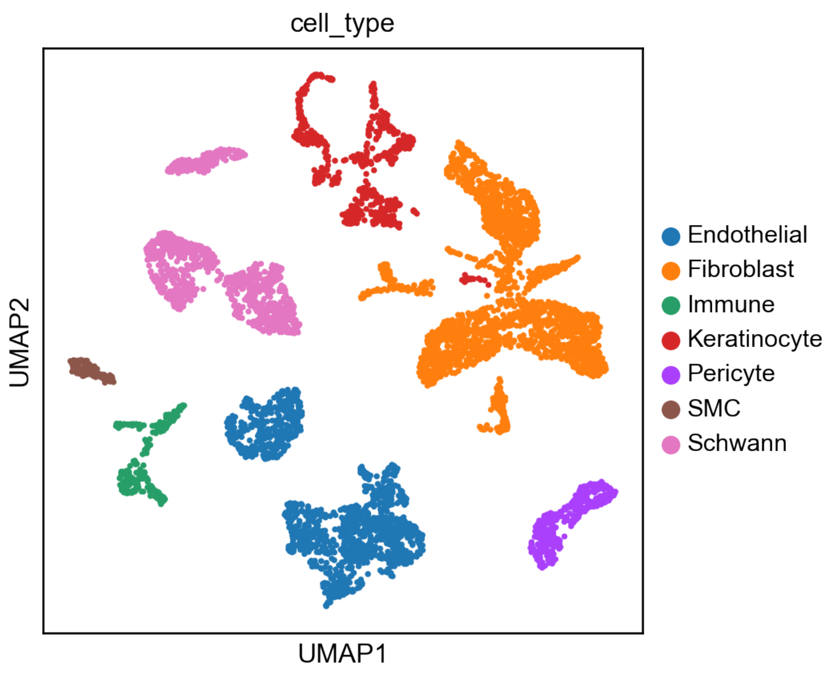

[38]:
```
sc.pl.umap(adata[adata.obs.cell_type=='Fibroblast'], color='leiden')
```

```
Trying to set attribute `.uns` of view, copying.
```

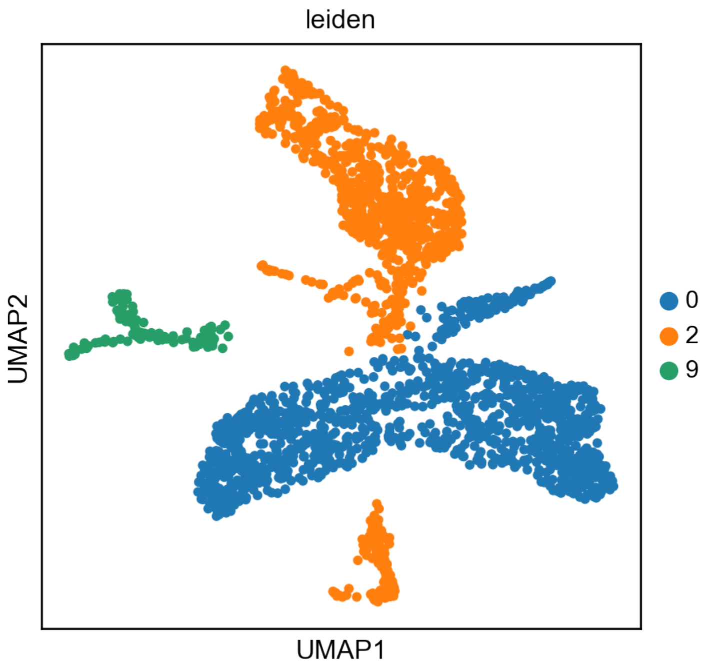

[39]:
```python
fbs = adata[adata.obs.cell_type=='Fibroblast']
```

[40]:
```python
fbs.write('P21 fibroblasts.h5ad', compression='lzf')
```

[41]:
```python
# delete all old attributes so we can recompute umap
del fbs.uns['neighbors']
del fbs.uns['pca']
del fbs.obsp['distances']
del fbs.obsp['connectivities']
del fbs.obsm['X_pca']
del fbs.obsm['X_umap']
del fbs.varm['PCs']


# compute umap embedding
sc.pp.neighbors(fbs, n_neighbors=10, n_pcs=40)
sc.tl.umap(fbs)
sc.pl.umap(fbs)
```

```
computing neighbors
WARNING: You're trying to run this on 2142 dimensions of `.X`, if you really
want this, set `use_rep='X'`.
         Falling back to preprocessing with `sc.pp.pca` and default params.
computing PCA
    with n_comps=50
    finished (0:00:00)
    finished: added to `.uns['neighbors']`
    `.obsp['distances']`, distances for each pair of neighbors
    `.obsp['connectivities']`, weighted adjacency matrix (0:00:00)
computing UMAP
    finished: added
    'X_umap', UMAP coordinates (adata.obsm) (0:00:04)
```

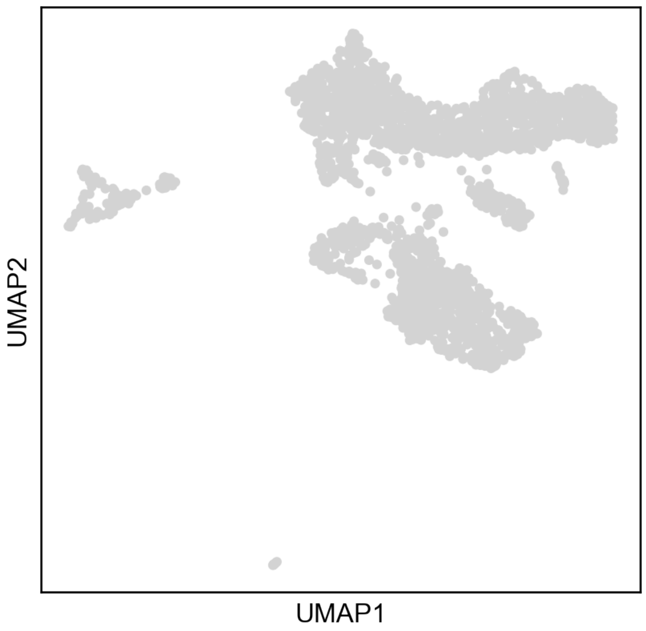

[47]:
```
# cluster the data
sc.tl.leiden(fbs, resolution=0.08)
sc.pl.umap(fbs, color=['leiden'])
```

```
running Leiden clustering
    finished: found 7 clusters and added
    'leiden', the cluster labels (adata.obs, categorical) (0:00:00)
```

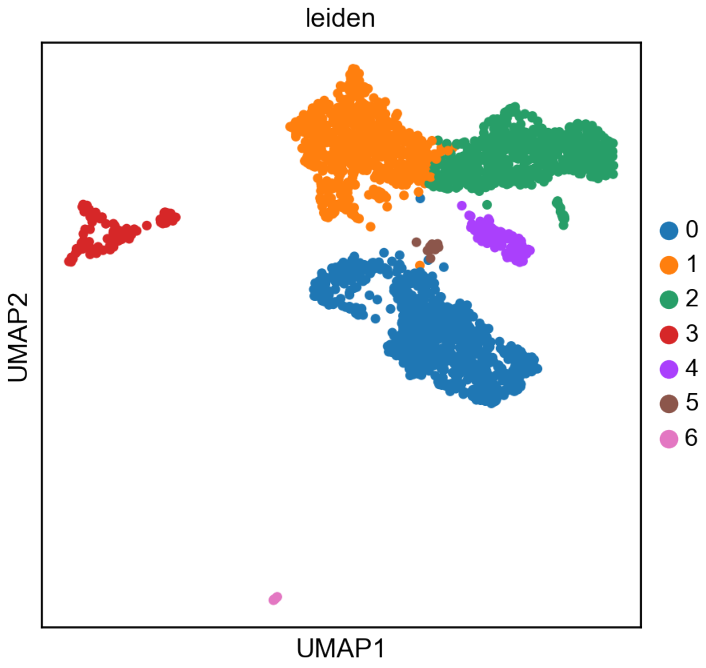

[48]:
```
sc.pl.umap(fbs, color=['leiden','Itga8','Ebf2'])
```

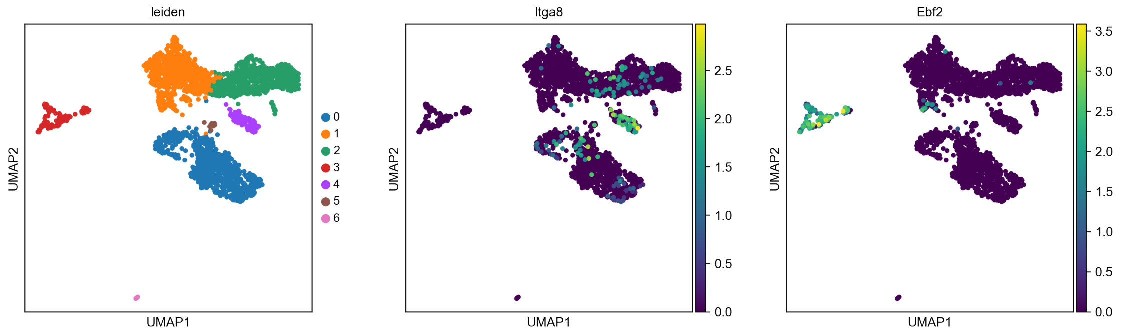

[50]:
```
sc.tl.rank_genes_groups(fbs, 'leiden', method='t-test')
pd.DataFrame(fbs.uns['rank_genes_groups']['names']).head(10)
```

```
ranking genes
    finished: added to `.uns['rank_genes_groups']`
    'names', sorted np.recarray to be indexed by group ids
    'scores', sorted np.recarray to be indexed by group ids
    'logfoldchanges', sorted np.recarray to be indexed by group ids
    'pvals', sorted np.recarray to be indexed by group ids
    'pvals_adj', sorted np.recarray to be indexed by group ids (0:00:00)
```

[50]:

	0	1	2	3	4	5	6
0	Igfbp3	Col3a1	Coch	Gsn	Igfbp4	Krt18	Rsrp1
1	Malat1	Clec3b	Tnmd	Igfbp6	Thy1	Krt8	Dct
2	a	Lum	Ogn	Ebf2	Aqp1	Krt19	Mitf
3	Inhba	Col1a1	Fmod	Mfap5	Vcan	Epcam	Mt2
4	Gpx1	Eln	Col11a1	Mgp	Nrp1	Ly6e	Gstp1
5	Fgfr1	Col1a2	Kera	Cav1	Tcf4	Tm4sf1	Mlana
6	Socs3	Sparc	Emid1	Cldn1	Col6a2	Rpl26	Rpl19
7	Ptma	Mfap4	Crabp1	Tgfbi	Col6a1	Rps28	Tyrp1
8	Vim	Col5a1	Ecm2	Fth1	Chrm2	Cldn3	Ptgds
9	Serpine2	Dcn	Mafb	Nr2f2	Pmp22	Ndufa4	Chchd2

[52]:
```
sc.pl.dotplot(fbs, var_names=['Ebf2','Itga8', 'Acta2'], groupby='leiden')
```

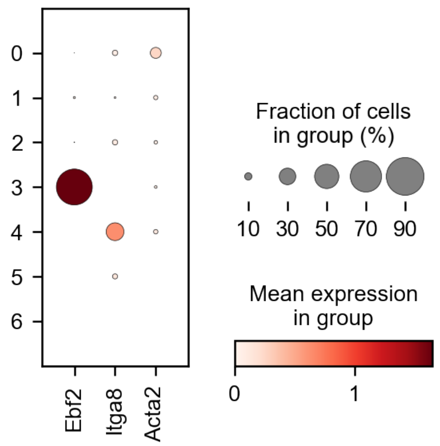

[63]:
```
cluster_names = ['DP/DS','Reticular','Coch','Ebf2','APM', '','']
```

[64]:
```
fbs.obs['cell_type'] = [cluster_names[int(x)] for x in fbs.obs.leiden]
```

Trying to set attribute `.obs` of view, copying.

[60]:
```
# Exclude very small undefined populations expressing keratinocyte and␣
↪melanocyte markers
fbs = fbs[fbs.obs.cell_type.isin(['DP/DS','Reticular','Coch','Ebf2','APM'])]
```

[65]:
```
sc.pl.umap(fbs, color=['cell_type'])
```

C:\Users\hhy696\OneDrive - Queen Mary, University of
London\Bioinformatics\bioinf\lib\site-packages\anndata_core\anndata.py:1220:

```
FutureWarning: The `inplace` parameter in pandas.Categorical.reorder_categories
is deprecated and will be removed in a future version. Removing unused
categories will always return a new Categorical object.
  c.reorder_categories(natsorted(c.categories), inplace=True)
… storing 'cell_type' as categorical
```

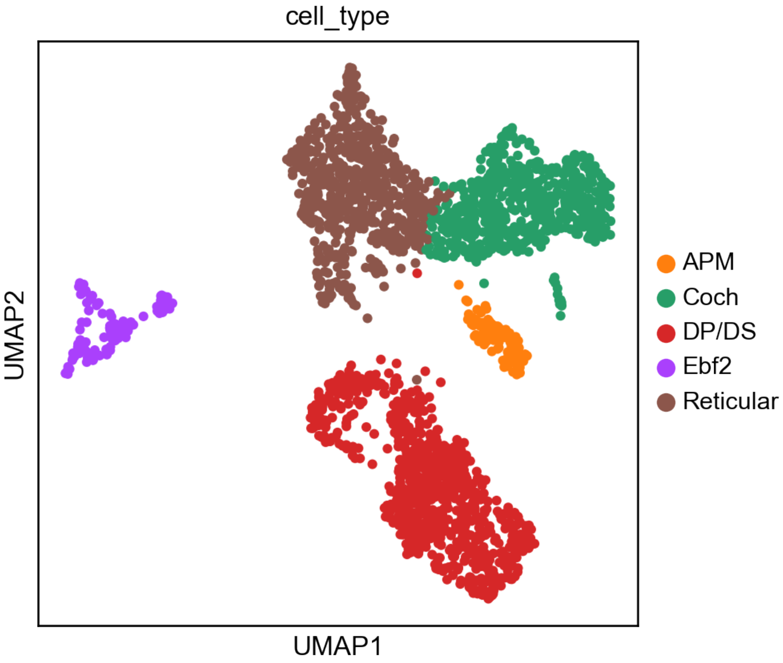

[69]:
```
sc.pl.umap(fbs, color=['Coch','Ly6a','Ebf2','Itga8','Fgf7','Fgf10'])
# Reticular: Ly6a
# APM: Itga8
# DP: Fgf7, Fgf10, Sox2
```

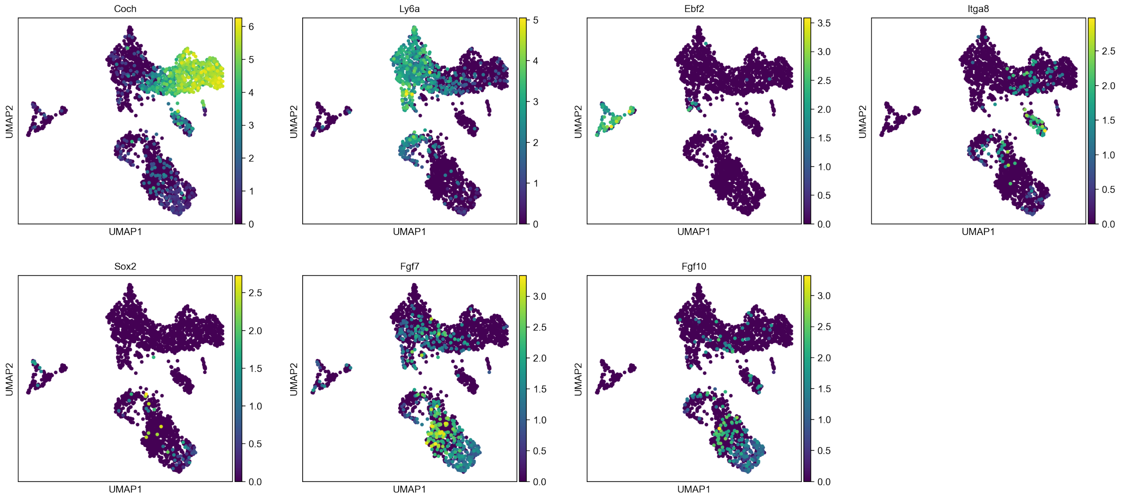

```
[70]: fbs.write('P21 fibroblasts.h5ad', compression='lzf')
```

```
[71]: sc.tl.rank_genes_groups(fbs, 'cell_type', method='t-test')
      pd.DataFrame(fbs.uns['rank_genes_groups']['names']).head(10)
```

```
ranking genes
    finished: added to `.uns['rank_genes_groups']`
    'names', sorted np.recarray to be indexed by group ids
    'scores', sorted np.recarray to be indexed by group ids
    'logfoldchanges', sorted np.recarray to be indexed by group ids
    'pvals', sorted np.recarray to be indexed by group ids
    'pvals_adj', sorted np.recarray to be indexed by group ids (0:00:00)
```

[71]:

	APM	Coch	DP/DS	Ebf2	Reticular
0	Igfbp4	Coch	Igfbp3	Gsn	Col3a1
1	Thy1	Tnmd	Malat1	Igfbp6	Clec3b
2	Aqp1	Ogn	a	Ebf2	Lum
3	Vcan	Fmod	Inhba	Mfap5	Col1a1
4	Nrp1	Col11a1	Gpx1	Mgp	Eln
5	Tcf4	Kera	Fgfr1	Cav1	Col1a2
6	Col6a2	Emid1	Ptma	Cldn1	Sparc
7	Col6a1	Crabp1	Lgals3	Tgfbi	Mfap4
8	Chrm2	Ecm2	Socs3	Nr2f2	Col5a1
9	Pmp22	Mafb	Vim	Fth1	Dcn

```
[78]: sig_genes = sc.get.rank_genes_groups_df(fbs, group=None, pval_cutoff=0.05,␣
      ↪log2fc_min=1)
```

```
[79]: sig_genes
```

[79]:

	group	names	scores	logfoldchanges	pvals	pvals_adj
0	APM	Igfbp4	22.838535	3.363599	2.985981e-45	7.258919e-43
1	APM	Thy1	14.598826	2.558907	2.126822e-27	1.731681e-25
2	APM	Aqp1	14.269781	3.620008	5.803036e-26	4.238208e-24
3	APM	Vcan	13.106678	3.801002	2.460013e-23	1.500431e-21
4	APM	Nrp1	12.734237	4.321249	1.753802e-22	9.882266e-21
…	…	…	…	…	…	…
3933	Reticular	Pigz	2.440641	3.167039	1.491760e-02	4.755077e-02
3934	Reticular	Bdkrb2	2.436770	2.448219	1.507234e-02	4.800411e-02
3935	Reticular	Prrt4	2.435076	23.212461	1.516508e-02	4.825819e-02
3936	Reticular	Mcoln2	2.433280	2.961850	1.521281e-02	4.837907e-02
3937	Reticular	C1rl	2.424847	1.227175	1.552969e-02	4.929466e-02

[3938 rows x 6 columns]

[80]:
```
ap = sc.queries.enrich(list(sig_genes[sig_genes.group=='APM'].names.values),␣
↪org='mmusculus', gprofiler_kwargs={'all_results':True, 'sources':['GO:BP']})
dpds = sc.queries.enrich(list(sig_genes[sig_genes.group=='DP/DS'].names.
↪values), org='mmusculus', gprofiler_kwargs={'all_results':True, 'sources':
↪['GO:BP']})
ebf = sc.queries.enrich(list(sig_genes[sig_genes.group=='Ebf2'].names.values),␣
↪org='mmusculus', gprofiler_kwargs={'all_results':True, 'sources':['GO:BP']})
coch = sc.queries.enrich(list(sig_genes[sig_genes.group=='Coch'].names.values),␣
↪org='mmusculus', gprofiler_kwargs={'all_results':True, 'sources':['GO:BP']})
ret = sc.queries.enrich(list(sig_genes[sig_genes.group=='Reticular'].names.
↪values), org='mmusculus', gprofiler_kwargs={'all_results':True, 'sources':
↪['GO:BP']})
```

[81]:
```
results = [ap,dpds,ebf,coch,ret]
for result in results:
    print(result[result.name.str.contains('response to UV')].name, end=' ')
    print(result[result.name.str.contains('response to UV')].p_value)
```

```
1795    cellular response to UV-A
1796    cellular response to UV-B
1901             response to UV-A
2274               response to UV
2354             response to UV-B
3407      cellular response to UV
Name: name, dtype: object 1795    1.0
1796    1.0
1901    1.0
2274    1.0
2354    1.0
3407    1.0
Name: p_value, dtype: float64
974     cellular response to UV-C
1364    cellular response to UV-B
4788             response to UV-C
4789             response to UV-B
4801               response to UV
8643      cellular response to UV
Name: name, dtype: object 974     1.0
1364    1.0
4788    1.0
4789    1.0
4801    1.0
8643    1.0
Name: p_value, dtype: float64
996           response to UV-A
3694             response to UV
6644    cellular response to UV
```

```
Name: name, dtype: object 996     1.0
3694    1.0
6644    1.0
Name: p_value, dtype: float64
649                 response to UV-A
2596    cellular response to UV-C
2597    cellular response to UV-B
3536                response to UV-C
3537                response to UV-B
3567                  response to UV
4720      cellular response to UV
Name: name, dtype: object 649     1.0
2596    1.0
2597    1.0
3536    1.0
3537    1.0
3567    1.0
4720    1.0
Name: p_value, dtype: float64
733                 response to UV-A
2899    cellular response to UV-B
2900    cellular response to UV-A
3453                  response to UV
4017                response to UV-B
5144      cellular response to UV
Name: name, dtype: object 733     1.0
2899    1.0
2900    1.0
3453    1.0
4017    1.0
5144    1.0
Name: p_value, dtype: float64
```

[82]:
```python
dpds[dpds.name.str.contains('response to UV')]
```

[82]:

	source	native	name	p_value	significant \
974	GO:BP	GO:0071494	cellular response to UV-C	1.0	False
1364	GO:BP	GO:0071493	cellular response to UV-B	1.0	False
4788	GO:BP	GO:0010225	response to UV-C	1.0	False
4789	GO:BP	GO:0010224	response to UV-B	1.0	False
4801	GO:BP	GO:0009411	response to UV	1.0	False
8643	GO:BP	GO:0034644	cellular response to UV	1.0	False

	description	term_size \
974	"Any process that results in a change in state…	6
1364	"Any process that results in a change in state…	9
4788	"Any process that results in a change in state…	14

```
4789  "Any process that results in a change in state…        18
4801  "Any process that results in a change in state…       148
8643  "Any process that results in a change in state…        89

      query_size  intersection_size  effective_domain_size  precision  \
974         1346                  2                  21118   0.001486
1364        1346                  2                  21118   0.001486
4788        1346                  4                  21118   0.002972
4789        1346                  4                  21118   0.002972
4801        1346                 19                  21118   0.014116
8643        1346                 11                  21118   0.008172

        recall    query                   parents
974   0.333333  query_1  [GO:0010225, GO:0034644]
1364  0.222222  query_1  [GO:0010224, GO:0034644]
4788  0.285714  query_1              [GO:0009411]
4789  0.222222  query_1              [GO:0009411]
4801  0.128378  query_1              [GO:0009416]
8643  0.123596  query_1  [GO:0009411, GO:0071482]
```

[83]:

```
names = ['ap','dpds','ebf','coch','ret']
for i in range(len(names)):
    results[i].to_csv('csv/p21/'+names[i]+'.csv')
```

[ ]:

```

```
