# Supplementary material for: Role of distinct fibroblast lineages and immune cells in dermal repair following UV radiation-induced tissue damage
Source: eLife. 2021 Dec 23;10:e71052. doi: 10.7554/eLife.71052 (PMC8747514; doi:10.7554/eLife.71052)
Supplement: Supplementary file 1. [file elife-71052-supp1.docx]

**Supplementary Table 1. Immune cell flow cytometry antibody panel for T cells and myeloid cells**

| **T cell Panel** |  |  |
| --- | --- | --- |
| **Laser/Filter** | **Dye** | **Antigen** |
| YG 582/15 | PE | CTLA-4 |
| YG 780/60 | PE-Cy7 | Ki67 |
| Blue 710/50 | PercP-Cy5.5 | TCRgd GL3 |
| Red 730/45 | A700 | CD45 |
| Red 780/60 | A780 | CD25 |
| Violet 610/20 | BV605 | CD8 |
| Violet 660/20 | BV650 | CD4 |
| Violet 710/50 | BV711 | CD3 |
| Violet 525/60 | Aqua LIVE/DEAD | GHOST |
| Violet 450/50 | eFluor 450 | Foxp3 |

| **Myeloid Panel** |  |  |
| --- | --- | --- |
| **Laser/Filter** | **Dye** | **Antigen** |
| YG 660/20 | PE-Cy5 | F4/80 |
| YG 582/15 | PE | MHCII |
| Red 780/60 | APC/Cy7 | Ly-6A/E (Sca-1) |
| Blue 530/30 | A488 | CD11c |
| Red 670/14 | A647 | CD11b |

| **Antibodies** | **Dilution** | **Manufacturer** | **Catno.** |
| --- | --- | --- | --- |
| Anti-Mouse/Rat Foxp3 eFluor450, FJK-16s | 1:100 | eBioscience | 48-5773-82 |
| Anti-Mouse CD152 (CTLA4) PE, UC10-4F10-11 | 1:100 | BD | 553720 |
| Anti-Human Ki67 PE-Cy7, B56 | 1:100 | BD | 561283 |
| Anti-Mouse TCR gd PerCP-Cy 5.5, GL3 | 1:300 | Biolegend | 118117 |
| Anti-Mouse CD45 Alexa Fluor700, 30-F11 | 1:200 | eBioscience | 56-0451-82 |
| Anti-Mouse CD25 APC-eFluor780, PC61.5 | 1:150 | eBioscience | 47-0251-82 |
| Brilliant Violet 605 anti-mouse CD8a, 53-6.7 | 1:200 | Biolegend | 100743 |
| Brilliant Violet 650 anti-mouse CD4 Antibody, RM4-5 | 1:200 | Biolegend | 100545 |
| Brilliant Violet 711 anti-mouse CD3 Antibody, 17A2 | 1:150 | Biolegend | 100241 |
| Ghost DyeTM Violet 510 Live/Dead Stain | 1:500 | Tonbo Biosciences | 13-0870-T100 |
| Anti-mouse CD11b (M1/70) AlexaFluor647 | 1:400 | Biolegend | 101220 |
| F4/80 Monoclonal Antibody (BM8), PE-Cy5 | 1:400 | eBioscience | 15-4801-80 |
| MHC Class II (I-A) Monoclonal Antibody (NIMR-4), PE | 1:500 | eBioscience | 12-5322-81 |
| APC/Cy7 anti-mouse Ly-6A/E (Sca-1) | 1:400 | Biolegend | 108125 |
| AlexaFluor488 anti-mouse CD11c | 1:400 | Biolegend | 117311 |
